# Specifically modified Env immunogens activate B-cell precursors of broadly neutralizing HIV-1 antibodies in transgenic mice

**DOI:** 10.1038/ncomms10618

**Published:** 2016-02-24

**Authors:** Andrew T. McGuire, Matthew D. Gray, Pia Dosenovic, Alexander D. Gitlin, Natalia T. Freund, John Petersen, Colin Correnti, William Johnsen, Robert Kegel, Andrew B. Stuart, Jolene Glenn, Michael S. Seaman, William R. Schief, Roland K. Strong, Michel C. Nussenzweig, Leonidas Stamatatos

**Affiliations:** 1 Vaccine and Infectious Disease Division, Fred Hutchinson Cancer Research Center, 1100 Fairview Avenue N, PO Box 19024 Seattle, Washington 98109, USA; 2 Laboratory of Molecular Immunology, New York, New York 10065, USA; 3 Center for Virology and Vaccine Research, Beth Israel Deaconess Medical Center, 3 Blackfan Circle, E/CLS-1001, Boston, Massachusetts 02215, USA; 4 Department of Immunology and Microbial Science, The Scripps Research Institute, 10550 North Torrey Pines Road La Jolla, California 92037, USA; 5 IAVI Neutralizing Antibody Center, The Scripps Research Institute, 10550 North Torrey Pines Road La Jolla, California 92037, USA; 6 Center for HIV/AIDS Vaccine Immunology and Immunogen Discovery, The Scripps Research Institute, 10550 North Torrey Pines Road La Jolla, California 92037, USA; 7 Ragon Institute of MGH, MIT, and Harvard, 400 Technology Square Cambridge, Massachusetts 02139, USA; 8 Howard Hughes Medical Institute, The Rockefeller University, 1230 York Avenue, New York, New York 10065, USA; 9 University of Washington, Department of Global Health, 1510 San Juan Road #310e Seattle, Washington 98195, USA

## Abstract

VRC01-class broadly neutralizing HIV-1 antibodies protect animals from experimental infection and could contribute to an effective vaccine response. Their predicted germline forms (gl) bind Env inefficiently, which may explain why they are not elicited by HIV-1 Env-immunization. Here we show that an optimized Env immunogen can engage multiple glVRC01-class antibodies. Furthermore, this immunogen activates naive B cells expressing the human germline heavy chain of 3BNC60, paired with endogenous mouse light chains *in vivo*. To address whether it activates B cells expressing the fully humanized gl3BNC60 B-cell receptor (BCR), we immunized mice carrying both the heavy and light chains of gl3BNC60. B cells expressing this BCR display an autoreactive phenotype and fail to respond efficiently to soluble forms of the optimized immunogen, unless it is highly multimerized. Thus, specifically designed Env immunogens can activate naive B cells expressing human BCRs corresponding to precursors of broadly neutralizing HIV-1 antibodies even when the B cells display an autoreactive phenotype.

## Abstract (web summary)

The induction of broadly neutralizing antibodies (bNAbs) is a goal of HIV-1 vaccine research. Here the authors demonstrate the ability of an HIV Env-derived immunogen to bind germline precursors of a class of bNAbs and to activate the corresponding B cells in a knock-in mouse model

## Graphical abstract

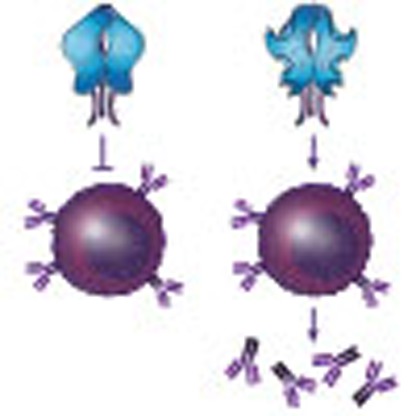

HIV-1 broadly neutralizing antibodies (bNAbs) target four major areas of the viral envelope glycoprotein (Env): the membrane proximal external region of the gp41 subunit; the CD4 receptor-binding site (CD4-BS), two sites comprising both carbohydrate and amino acid moieties, one at the base of the V3 and another on the V1/V2 loops of the gp120 subunit; and more recently, bNAbs have been identified that target complex regions spanning elements of both gp120 and gp41 (refs 1, 2).

On the basis of their ontogenies and mode of recognition, the CD4-BS bNAbs are grouped into two major types: heavy-chain complementary determining region three (CDRH3)-dominated and variable heavy (VH)-gene-restricted^3^. Antibodies that make contact primarily through their CDRH3 regions are further subdivided into the CH103, HJ16, VRC13 and VRC16 classes, while the VH-gene-restricted Abs include the VRC01- and the 8ANC131-class antibodies (derived from VH1-2 and VH1-46, respectively), which make contact primarily through their CDRH2 domains.

VRC01-class bnAbs protect non-human primates from experimental SHIV-infection^4^,^5^,^6^,^7^ and humanized mice from HIV-1 infection^8^,^9^. It is, therefore, likely that vaccine-elicited VRC01-class bNAbs will protect humans from HIV-1 infection. With the exception of llamas that express heavy-chain-only antibodies^10^, all efforts to elicit such antibodies by immunization in humans or wild-type animals with recombinant Env have been unsuccessful. One of the many important reasons for this is thought to be the inability of the Env proteins used as immunogens to engage B-cell receptors (BCRs) that encode the germline (gl) of VRC01-class antibodies^11^,^12^,^13^. Indeed, maturation of these antibodies to full neutralizing activity requires that they circumvent steric constraints on Env through extensive somatic hypermutation^3^,^14^,^15^,^16^.

HIV-1 has evolved to avoid detection by the progenitor B cells that give rise to VRC01-class bNAbs through the selection of specific N-linked glycosylation sites (NLGS) in Loop D and V5 of the gp120 subunit of Env^12^,^13^. As a consequence, recombinant Env proteins derived from diverse HIV-1 isolates are ineffective in binding to and stimulating B cells engineered to express the glBCR forms of VRC01-class bNAbs *in vitro*^12^,^13^. We have shown that targeted disruption of conserved NLGS at position 276 in Loop D, and at positions 460 and 463 in V5 of the 426c clade C Env, permits binding and activation of B cell lines expressing glBCRS of two clonally related VRC01-class bNAbs, VRC01 and NIH45-46 *in vitro*^13^. These two BCRs represent a small subset of potential VRC01-class antibody progenitors. Thus, designing immunogens capable of recognizing a larger group of glVRC01-class BCRs should increase the chances of activating rare^17^, naive glVRC01-class B cells during human immunization.

Here we describe the identification of specific modifications that expand the glVRC01-class antibody-recognition potential of the 426c Env. One such Env was shown to activate B cells in mice in which the gl3BNC60 VH alone was knocked-in ref. 18. However, it is not known whether this same immunogen would be able to activate B cells expressing the fully human gl3BNC60 BCR *in vivo*. To address this point, we immunized knock-in mice, homozygous for the VH and VL of gl3BNC60 (gl3BNC60 KI). Similar to other anti-HIV-1 antibody knock-in mice^19^,^20^,^21^, gl3BNC60 B cells displayed a phenotype consistent with anergy^22^,^23^ and were inefficiently activated by soluble trimeric forms of our modified 426c immunogen. Nevertheless, higher-order multimeric forms of the immunogen stimulated the proliferation of gl3BNC60KI B cells in these mice indicating that this potential barrier to HIV-1 vaccination can be overcome by specific immunogen design.

## Results

### NLGS disruption is not a panacea for glVRC01 class binding

**Figure 1:**
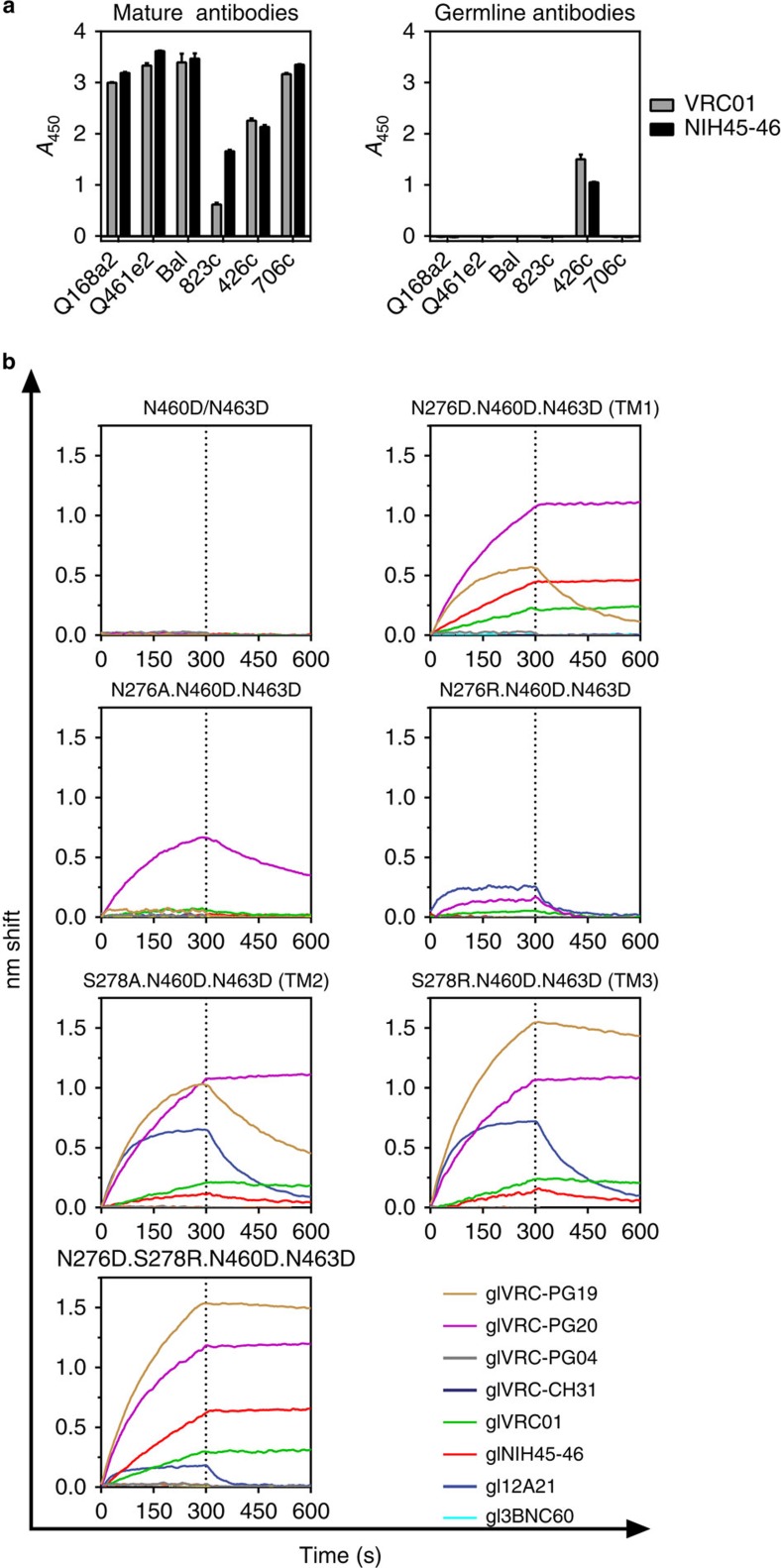
Germline VRC01-class antibody-binding to 426c gp120 variants. (**a**) ELISA was used to evaluate the binding of mature (left panel) or germline (right panel) VRC01 and NIH45-46 to the indicated Envs lacking NLGS in Loop D and V5. Error bars represent the s.d. from three technical replicates. (**b**) His-tagged gp120 variants of 426c were immobilized on a Ni-NTA biosensor and binding to 20 μg ml^−1^ solution of the indicated germline, VRC01 class antibodies was measured by BLI. The mutations tested are indicated on the top of each panel (nomenclature in parentheses corresponds to that in Table 1). BLI traces are representative of at least three independent experimental replicates. Dotted line demarcates the association and dissociation phases.

**Figure 2:**
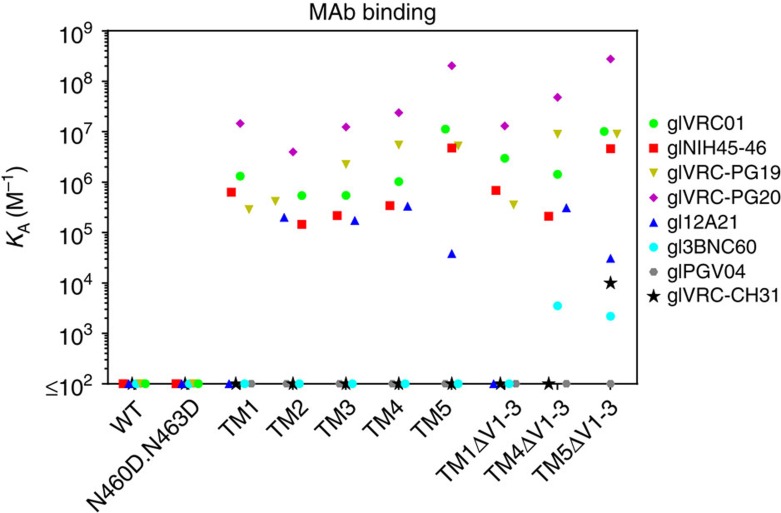
Binding affinities of the indicated germline VRC01-class antibodies to selected 426c variants. Soluble trimeric 426c gp140 variants were biotinylated and immobilized on a streptavidin biosensor. The association constant of the various germline VRC01-class antibodies was determined by BLI, as described in the Methods section. Undetectable antibody-Env binding is shown on the *x* axis. Full kinetic parameters are shown in Supplementary Table 1. See Table 1 for a description of the various mutations on the 426c Env.

The disruption of the NLGS at position 276 (N276D) from the clade C recombinant Env 426c, results in glVRC01 and glNIH45-46 antibody-binding^13^. The parallel disruption of two NLGS in V5 (positions 460 and 463) improves this binding; although by themselves N460D and N463D are insufficient to confer glVRC01- or glNIH45-46-binding^13^. We term the 426c Env lacking these three NLGS as ‘triple mutant 1′ (TM1). The disruption of the 276 and V5 NLGS from other Envs does not result in glVRC01-binding (Fig. 1a) indicating that additional constraints are present on these Envs that prevent glVRC01-binding. Because of its ability to engage certain glVRC01-class BCRs *in vitro*, TM1 is a logical immunogen to target naive B cells expressing glVRC01-class BCRs *in vivo*. Here we report that TM1 is also recognized by two additional, clonally related, glVRC01-class antibodies, glVRC-PG19 and glVRC-PG20 (Fig. 1b and Supplementary Table 1). However, other germline-reverted members of the VRC01-class antibodies such as: 12A21, 3BNC60, VRC-CH31 or VRC-PG04 do not bind TM1 (Figs 1 and 2 and Supplementary Table 1).

### Nature of 276 NLGS disruption affects glVRC01-class binding

The reasons for the differential recognition of TM1 by the different glVRC01-class antibodies are not well understood. Information on this topic will help to better understand the features of Env that prevent the engagement of the various glVRC01-class BCRs in the context of HIV-1 infection and will facilitate the design of immunogens capable of activating a broader range of these receptors.

Although the VH domains of all known VRC01-class bNAbs are derived from the VH1-2*02 allele and a few J genes, their CDRH3 regions differ extensively in amino-acid sequence and length^3^,^24^,^25^,^26^,^27^. Therefore, one possible explanation for the differential recognition of TM1 by different members of the glVRC01-class antibodies may be due to the different CDRH3 regions expressed by these antibodies. It is also possible that the above-mentioned differences in TM1 recognition by the various glVRC01-class antibodies is linked to the different light chains (LCs) used by these antibodies. Relevant to the latter possibility is the observation that when the germline heavy chains (glHCs) of 3BNC60 and gl12A21 are paired with the germline light chains (glLC) of VRC01/NIH45-46, the chimeric antibodies bind TM1 (ref. 13). These results suggest that the glLCs of 12A21 and 3BNC60 (derived from *Vκ1-33* while those of VRC01/NIH45-46 are derived from *Vκ3-11*) are incompatible with TM1-binding. The N to D substitutions used to generate TM1, were initially selected to maintain structural similarity at positions 276, 460 and 463. However, they introduce negative charges at those positions; which potentially create unfavourable electrostatic interactions with the more negatively charged CDRLs on the glLCs of 12A21 and 3BNC60 (as compared with the CDRL regions of the VRC01/NIH45-46 glLC; Supplementary Fig. 1). We therefore introduced different amino acids at position 276 and examined their effect on glAb-binding (Fig. 1b and Supplementary Table 1). On the background of the 426c.N460D.N463D, which does not bind glVRC01-class antibodies (Fig. 1b), the substitution of N276 either by an alanine, which introduces a neutral charge, or by an arginine, which introduces a positive charge, results in a drastic reduction in binding of glVRC01, glNIH45-46, glVRC-PG19 and glVRC-PG20 relative to TM1. However, the N276R mutation conferred weak but detectable binding to gl12A21 (Fig. 1b). We concluded that the nature of the amino acid at position 276 influences the interactions between 426c and glVRC01 class bNAbs. Specifically, a negative charge at position 276 may be favoured over a neutral or positive charge by glVRC01, glNIH45-46, glVRC-PG19 and glVRC-PG20, but that the negative charge is incompatible with gl12A21-binding.

Alternatively, we disrupted the NLGS at position 276 by mutating S278. Interestingly, a S278A substitution (TM2) conferred gl12A21 binding, while it reduced binding of glNIH45-46, glVRC01 and glVRC-PG20, and improved binding of glVRC-PG19 compared with TM1 (although this Ab still had a relatively fast off-rate; Fig. 1b and Supplementary Table 1). A S278R substitution (TM3) had a similar overall effect as the S278A substitution, but the binding of glVRC-PG20 and glVRC-PG19 was improved, the latter having a slower off-rate than the one recorded with the S278A mutation compared with TM1 (Fig. 1b and Supplementary Table 1). The combination of N276D and S278R conferred weak gl12A21-binding, but improved glNIH45-46-binding (Fig. 1b) compared with TM1. We concluded that gl12A21 displays preferential binding to 426c variants with an N at position 276. In contrast, glVRC-PG19, glVRC01 and glNIH45-46 can accommodate either an N or D at that position. Thus, the absence of an NLGS at position 276 is important for glVRC01, glNIH45-46, glVRC-PG19/20 and gl12A21 binding, but the specific amino acid requirements at positions 276 and 278 differ among these antibodies. Binding of gl3BNC60, glVRC-CH31 and glVRC-PGV04 was not recorded to any of these modified Envs (Figs 1 and 2 and Supplementary Table 1).

In addition to the S278R modification discussed above, Jardine *et al*., identified a combination of five additional amino acid mutations (L260F, K357R, I371F, N386D and G471S) which on the background of the engineered outer domain of eOD-base (which also contains the N276D and the N463D modifications) resulted in eOD-GT6 that bound glVRC01, glNIH45-46, glVRC-PG19/20 and gl12A21, and to gl3BNC60, glVRC-CH31 and glVRC-PG04 (ref. 12). eOD-GT6 is derived from HxB2 which is a CXCR4-tropic tier 1 virus while TM1 is a CCR5-tropic tier 2 virus. Furthermore, TM1 is comprised of the outer and inner domains of gp120, while eOD-GT6 is designed to recapitulate only the outer domain structure of Env. Therefore, we assessed whether these additional mutations will improve binding of glVRC01 class Abs on the background of TM1 as they did on eOD-GT6. On the TM1 background, the combination of these six mutations abrogated the binding of all glVRC01-class antibodies tested (Supplementary Fig. 2 ‘TM1+eOD-GT6') as compared with TM1 (Supplementary Fig. 2, top left panel).When these mutations were introduced individually, the K357R and N386D mutations displayed similar binding to that of TM1, while the L260F mutation was found to be detrimental (Supplementary Fig. 2). The G471S mutation improved the binding of glVRC-PG19, and the I371F mutation improved the binding of glVRC-PG20 but reduced the binding of glVRC-PG19 (Supplementary Fig. 2). The S278R mutation was discussed above. The S278R+G471S combination resulted in slightly improved binding to that observed with the S278R mutation (Supplementary Fig. 2). In sum, binding of glVRC01, glNIH45-46, glVRC-PG19, glVRC-PG20 and gl12A21 was observed when the S278R or S278R+G471S mutations were introduced on the background of the N276D, N460D and N463D mutations (Supplementary Fig. 2; ‘TM5'). We measured the affinity of glVRC01-class antibody binding to selected trimeric variants of the Envs mentioned above (Supplementary Table 1). None of these mutations conferred binding to gl3BNC60, glVRC-PG04 or glVRC-CH31 (Fig. 2, Supplementary Table 1).

### Variable loops restrict access of some glVRC01-class Abs

The lengths and glycosylation patterns of variable regions 1, 2 and 3 of gp120 (V1, V2, V3, respectively) influence the accessibility of diverse mature anti-CD4-BS antibodies. We also reported that deletion of the V1, V2 and V3 from TM1 gp140 improved the activation of B cells expressing the glNIH45-46 BCR^28^. To investigate whether these regions restrict binding to glVRC01 class antibodies on various modified 426c constructs, we engineered versions of TM1, TM4 and TM5 lacking V1, V2 and V3 (ΔV1–3). We observed gl3BNC60-binding to TM4ΔV1–3 and TM5ΔV1–3 (Fig. 2 and Supplementary Table 1) while removal of the variable regions from TM5 conferred binding to glVRC-CH31. To define the role of the individual variable regions of Env in restricting the binding of the gl3BNC60 antibody, we engineered versions of TM4 lacking these regions individually, or in various combinations (Supplementary Fig. 3). Although the individual removal of V1, V2 or V3 did not result in detectable gl3BNC60-binding, the simultaneous removal of the V1 and V2 regions did result in such binding. The additional removal of V3 modestly improved gl3BNC60-binding. We conclude that with the exception of glVRC-PG04, the removal of V1/V2 from TM4 improves and expands its binding capabilities to glVRC01-class Abs and that the V1/V2 region imposes an additional constraint to gl3BNC60-binding compared with the other glVRC01-class Abs examined. We conclude therefore that despite the ‘similar' structural features and similar recognition patterns of mutated VRC01-class bNAbs^3^,^14^,^15^,^16^, the germline antibodies display distinct requirements for Env-binding, suggesting that the germline forms of these antibodies have different angles of approach for the CD4-BS.

### The gl3BNC60 BCR displays autoreactivity in KI mice

Mice, rats, rabbits and NHPs do not express a human VH1-2*02 orthologue and thus are not suitable to evaluate immunogens designed to stimulate glBCRs of VRC01-class Abs^12^,^29^,^30^. To study the activation of naive B cells expressing the predicted germline version of a VRC01-class Ab *in vivo*, we engineered knock-in mice homozygous for the VH and VL of gl3BNC60 (gl3BNC60-KI). Because gl3BNC60 has the lowest binding affinity for TM4ΔV1-3 among the glVRC01-class antibodies tested (Fig. 2 and Supplementary Table 1), we reasoned that *in vivo* activation of B cells expressing the gl3BNC60 BCR should also translate into the activation of B cells expressing the other glVRC01-class BCRs that display stronger binding to this Env (that is, glVRC01 or gl12A21).

**Figure 3:**
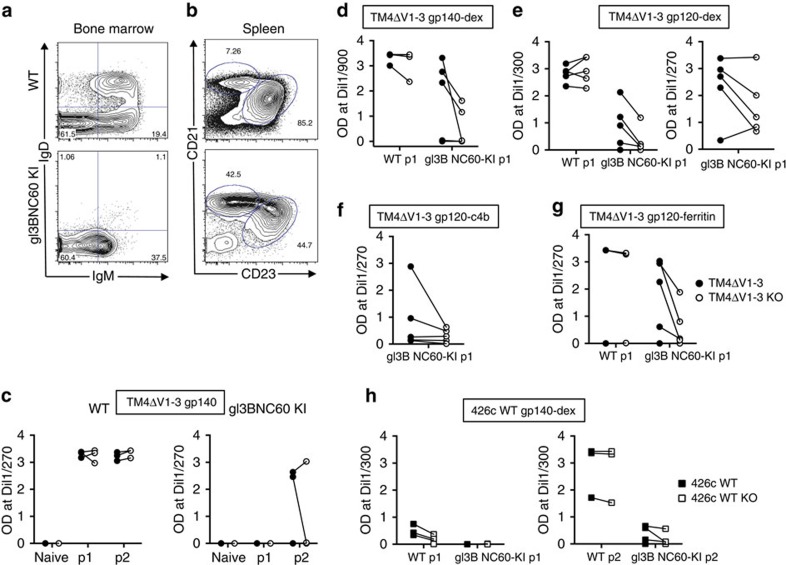
Antibody responses elicited in WT and knock-in gl3BNC60 mice after immunization. (**a**) Bone marrow cells (gated on; live cells, CD4^−^, CD8^−^, GR-1^−^ and B220^+^) of naive WT (top) and naive gl3BNC60 knock-in mice (bottom) were stained for IgD and IgM as indicated to identify immature (IgM^−/low^, IgD^−^), and mature (IgM^+^, IgD^+^) B-cell populations. (**b**) Splenocytes from naive WT and gl3BNC60 knock-in mice (gated on; live cells, CD4^−^, CD8^−^, GR-1^−^, B220^+^ and CD93^−^) were stained with CD21 and CD23 as indicated to identify follicular (CD21^low^/CD23^high)^ and marginal zone (CD21^high^/CD23^low^) B cells. Panels **a** and **b** show representative FACS diagrams from one individual mouse of five. (**c**) Serum IgG collected prior to (naive) and following one (p1) or two immunizations (p2) with 426cTM4ΔV1-V3 gp140 in Alum Imject were tested for binding to 426cTM4ΔV1-V3 gp140 (closed circles) or 426cTM4ΔV1-V3.gp140.D368R.E370A protein (KO; open circles) in WT (left panel, *n*=3) and knock-in gl3BNC60 mice (right panel, *n*=3) by ELISA. Lines connecting the black and white circles indicate OD values for 426cTM4ΔV1-V3 gp140 or 426cTM4ΔV1-V3.gp140.D368R.E370A proteins from the same mouse at the indicated concentrations. (**d**) Same as in **c** but WT mice (*n*=5) or knock-in gl3BNC60 (*n*=5) mice were immunized once with 426cTM4ΔV1-V3 gp140 dextramer in Alum Imject. (**e**) Same as in **c**, but WT (*n*=5) or knock-in gl3BNC60 mice (*n*=5) were immunized once with 426cTM4ΔV1-V3 gp120-dextramers in Alum Imject (left panel) or in Ribi adjuvant (*n*=5) (right panel). (**f**) Same as in **c,** but knock-in gl3BNC60 mice (*n*=5) were immunized once with 426c.TM4ΔV1-V3 gp120-C4b in Ribi adjuvant. (**g**) Same as in **c** but WT mice (*n*=3) or knock-in gl3BNC60 mice (*n*=5) were immunized once with 426cTM4ΔV1-V3 gp120-ferritin in Alum Imject. (**h**) Serum IgG from WT (*n*=4) and knock-in 3BNC60 mice (*n*=5) after one (left panel) or two (right panel) immunizations with WT 426c gp140-dextramer in Alum Imject were tested for binding to WT 426c gp140 (closed squares) or 426c.D368R.E370A (426c-KO, open squares) by ELISA. Lines between black and white squares indicate OD values for WT 426c gp140 or 426c-KO proteins from the same mouse at the indicated concentrations.

**Figure 4:**
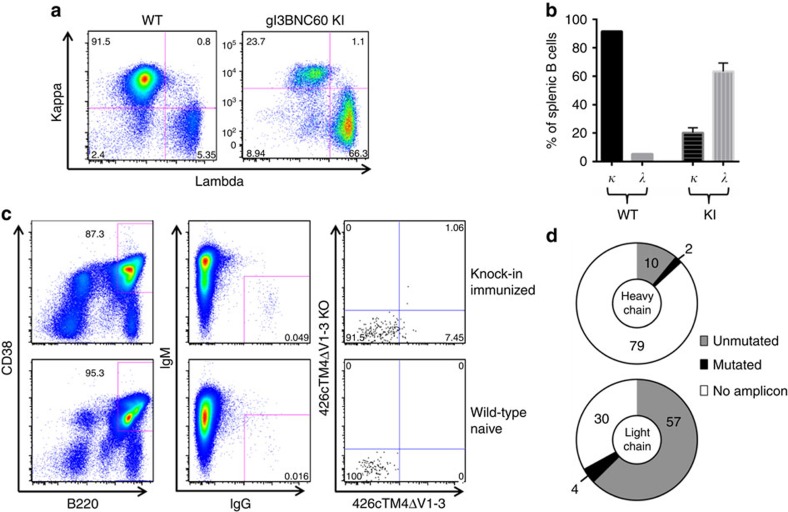
Sequencing of antibody and light chain transcripts from gl3BNC60 KI mice. (**a**) CD4^−^CD8^−^, Ly-6G^−^ and Ly-6C^−^ B220^+^ CD19^+^ splenocytes from WT (left panel) and gl3BNC60 KI (right panel) mice were stained with anti-mouse kappa, or lambda antibodies. (**b**) Summary of kappa and lambda frequencies in WT (*n*=1) and gl3BNC60 KI mice (*n*=6) as determined by counting the single kappa-positive or single lambda-positive populations in **a**. Error bars represent s.d. (**c**) Representative FACS plots of splenocytes from an immunized gl3BNC60 KI mouse (top panels) and a naive wild-type mouse (bottom panels). In brief, CD4^−^, CD8^−^, Gr1^−^ splenocytes were stained with the B-cell markers B220 and CD38 (left panel), B220^+^,CD38^+^ cells were stained for IgG and IgM (middle panel). Class switched IgM^−^ IgG^+^ cells were stained with 426c.TM4ΔV1-V3 and 426c.TM4ΔV1-V3-KO (right panel). 426c.TM4ΔV1-V3^+^, 426c.TM4ΔV1-V3-KO^−^ (lower right hand quadrant, of upper right panel) single cells from immunized mice were sorted into individual wells on a 96-well plate. (**d**) Summary of gl3BNC60 heavy- and light-chain sequences recovered from expressed transcripts. Numbers indicate the number of wells belonging to each category.

In contrast to WT mice, gl3BNC60 KI mice showed very few IgD^+^IgM^+^ B cells in the bone marrow (Fig. 3a) a phenotype displayed by mice with autoreactive B cells^22^,^23^, such as those expressing the mutated forms of the HIV-1 neutralizing antibodies 2F5 or 4E10 (refs 19, 20, 21). Thus, despite the fact that soluble gl3BNC60 IgG is not polyreactive^24^, this BCR is unable to support normal levels of B-cell development in knock-in mice. Although B-cell development was altered in gl3BNC60 KI mice, B cells survive and populate the spleen. Splenic B cells in the knock-in mice were skewed towards a marginal zone phenotype (CD21^high^, CD23^low^), in contrast to the WT, which were mostly follicular B cells (CD21^low^, CD23^high^; Fig. 3b). Consistent with the idea that the knock-in BCR is selected against, the majority of B cells in the spleen of the gl3BNC60 KI mice express an endogenous lambda light chain rather than the exogenous kappa light chain (Fig. 4a). Thus, only a small fraction of the B cells in gl3BNC60 KI mice express the fully human gl3BNC60 BCR.

### Immunization of gl3BNC60 KI mice with modified Env

WT or gl3BNC60 KI mice were immunized with the soluble gp140 trimeric form (gp140) of TM4ΔV1-3 Env (Fig. 3c and Supplementary Fig. 4a). Pre-immune serum IgG from the gl3BNC60 KI mice or the WT mice did not display reactivity to TM4ΔV1-3 Env. All the three WT animals produced Ag-specific IgG after a single immunization, while two immunizations were required for Ag-specific IgG production by the gl3BNC60 KI mice and only two of three animals responded. The relative inefficiency by which soluble trimeric gp140 TM4ΔV1-3 induces Ab production in the KI mice, might be related to its fast off-rate (Supplementary Table 1), or to B-cell anergy associated with autoreactivity^23^,^31^.

The ability of antigens with low binding affinities (due to fast off-rates) to activate B cells can be improved by increasing their valency^32^. Multimerizing an antigen can also overcome poor B-cell responses related to anergy in a manner that allows these B cells to receive T cell help and to produce somatically hypermutated BCRs and antibodies displaying no, or limited, autoreactivity^31^,^33^. To that end, the following multimerization approaches were tested with the TM4ΔV1–3 construct: (a) a dextran-based antigen-multimerization approach that can lead to up to 70 Env molecules per dextran molecule. This approach was previously used to stimulate B cells in knock-in mice expressing the germline 3BNC60 HC only (gl3BNC60 HC)^18^; (b) addition of the multimerization domain of the human C4b-binding protein to the carboxy (C) terminus of Env (this approach leads to the formation of ring-like structures expressing seven Env molecules)^34^; and (c) a ferritin-based approach, which leads to the formation of particles with 24 copies of Env^35^.

WT or gl3BNC60 KI mice were immunized with dextrameric gp140 (gp140-dex) (Fig. 3d and Supplementary Fig. 4b); dextrameric gp120 (gp120-dex) (Fig. 3e and Supplementary Fig. 4c); heptameric gp120 (gp120-C4b) (Fig. 3f and Supplementary Fig. 4d) and ferritin-gp120 (gp120-ferritin) (Fig. 3g and Supplementary Fig. 4e). The protein-based multimerization approaches show differences in size by size-exclusion chromatography (SEC) (Supplementary Fig. 5a) and BN-PAGE (Supplementary Fig. 5b). A single immunization with the multimerized Env constructs was sufficient to elicit immunogen-specific antibody responses (filled circles) in the majority (21 out of 24) of gl3BNC60 KI animals (3/5 in gp140-dex, 9/10 in gp120-dex, 5/5 in gp120-C4b and 4/5 in gp120-ferritin; Fig. 3d–g and Supplementary Fig. 4b–e, respectively). gl3BNC60 KI and WT mice were also immunized with dextrameric WT gp140 (Fig. 3h and Supplementary Fig. 4f). Three of four WT animals generated serum IgG responses to this immunogen (including CD4-BS antibodies) after a single immunization (Fig. 3h, left panel and Supplementary Fig. 4f). In contrast, none of the gl3BNC60 KI mice generated a detectable antibody response. A second immunization boosted the anti-Env antibodies in the WT mice and induced low responses in gl3BNC60 KI mice, but decreased the relative proportion of the CD4-BS directed antibodies in the WT response (Fig. 3h, right panel and Supplementary Fig. 4f). With the exception of one animal in the gp120-ferritin group (Fig. 3g and Supplementary Fig. 4e), the WT animals also responded after a single immunization (Fig. 3d–g and Supplementary Fig. 4b–e). We concluded that (i) the 426c modifications that are necessary for the binding of gl3BNC60 antibody to TM4ΔV1–3 lead to the stimulation of naive B cells expressing gl3BNC60 BCRs *in vivo*; and (ii) efficient stimulation of naive B cells expressing gl3BNC60-KI BCRs is facilitated by Env multimerization.

The presence of anti-CD4-BS Abs in immune sera from WT and gl3BNC60 KI mice was inferred by comparing the antibody titres to the immunogen (filled circles) with those against CD4-BS KO (D368R and E370A) version of the immunogen (open circles, Fig. 3c-g, and solid and dashed lines Supplementary Fig. 4a-f). The majority gl3BNC60 KI mice that responded to immunization with TM4ΔV1–3 generated anti-CD4-BS antibodies (19 out of 23: 1/2 in the gp140-immunized group; 3/3 in gp140-dex-immunized group; 4/4 in gp120-dex+Alum-immunized group; 3/5 in gp120-dex+Ribi-immunized group; 4/5 in gp120-C4b-immunized group and 4/4 in gp120-ferritin-immunized group), but the relative proportion of these Abs over the total vaccine-induced Abs varied depending on the animal rather than the multimerization platform used (dextramer versus ferritin particles, for example), or the form of the immunogen (gp120 versus gp140). Although WT animals immunized with TM4ΔV1–3 developed stronger antibody responses than the gl3BNC60 KI mice, only two WT animals (one immunized with gp140-dex and one immunized with gp120-dex) developed detectable, but low titres of anti-CD4-BS antibodies (Fig. 3d and e and Supplementary Fig. 4). We conclude that immunization with TM4ΔV1–3 stimulates the production of CD4-BS-specific antibody responses in the gl3BNC60 KI mice whereas WT gp140 does not.

### Isolation of B cells from immunized gl3BNC60 KI mice

In WT mice, 92% of naive B cells in the spleen express κLCs and only 5% express λLCs (Fig.4 a,b). In contrast only 20% of naive B cells present in the spleen express the exogenous human κLC in the gl3BNC60 KI mice. Following a single immunization with ferritin gp120, we isolated vaccine-specific anti-CD4-BS single B cells from gl3BNC60 KI splenocytes from two mice corresponding to the red and orange curves in Supplementary Fig. 4e (Fig. 4c). We amplified heavy and light chains from each individual cell using primers specific for the gl3BNC60-VH and the gl3BNC60-Vκ. Out of 91 wells tested, we successfully amplified gl3BNC60-VH from 12 wells, and amplified the gl3BNC60 Vκ from 61 wells (∼67%; Fig. 4d and Supplementary Table 2). Thus despite the observation that B cells expressing the endogenous gl3BNC60-Vκ are the minority in the spleen of these mice (Fig. 4a), the majority of Ag-specific B cells isolated express the exogenous human κLC (Fig. 4d, Supplementary Fig. 6 and Supplementary Table 2). Thus our optimized immunogen selects for B cells that express the exogenous gl3BNC60-Vκ.

Most of these HC and LC transcripts were unmutated, with two VH and three VL chains having 1–3 amino acid changes from germline, indicative of having undergone limited somatic hypermutation (Supplementary Fig. 6 and Supplementary Table 2). A fourth VL chain had undergone more extensive somatic hypermutation, resulting in eight amino acid changes from germline (Supplementary Fig. 6 and Supplementary Table 2). Consistent with this low rate of somatic hypermutation, serum IgG from gl3BNC60 KI mice immunized with gp120-dex, heptameric gp120 or ferritin-gp120 did not display HIV-1 neutralizing activity (Supplementary Table 3). The lack of elicitation of HIV-1 NAbs following a single immunization with TM4ΔV1–3 is likely due to the inability of the germline antibody to bind Env containing the variable loops, which are present on the viral Env spike. Higher levels of somatic hypermutation will likely be required to confer binding to full-length Env variants. A similar lack of neutralizing activity was recently reported in the above-mentioned gl3BNC60-VH KI mice immunized with TM4ΔV1–3 or with a multimeric, engineered gp120 outer domain (eOD-GT8-60mer)^18^ and in transgenic mice engineered to express the germline heavy chain of another VRC01-class antibody (VRC01) immunized with eOD-GT8-60mer (ref. 36).

## Discussion

Elicitation of VRC01-class bNAbs will likely require sequential immunizations with combinations of immunogens that expand naive B-cell progenitors of VRC01-class Abs followed by immunogens that further select for specific mutations after an initial expansion^12^,^15^,^37^,^38^. In support of this proposal, Dosenovic *et al*.^18^, recently reported that although specially modified germline-targeting immunogens, but not a native-like Env, were required to stimulate B cells expressing the gl3BNC60 HC, the elicitation of broadly neutralizing antibody responses in a mouse model expressing a synthetic intermediate of 3BNC60 required immunization with a native-like Env. In addition, viral and neutralizing antibody evolution data collected from longitudinal HIV-1 infection case studies, demonstrated that the development of bNAbs targeting either a complex epitope encompassing the V2 loop, CAP256-VRC26 (ref. 39), or the CD4-BS, CH103 (ref. 40), was dependent on the emergence of particular viral variants capable of activating the germline BCR forms of these antibodies and on the continuous evolution of variants capable of driving the mutation of BCRs along particular evolutionary pathways. Although, the precise evolutionary path(s) of VRC01-class bNAbs, and the co-evolving viruses during infection are not yet known (due to the unavailability of appropriate longitudinal samples), the available information strongly argues for a complex and prolonged evolution of the germline VRC01-class BCRs during chronic infection^26^.

Here we report the design of a clade C-derived Env that can bind a wide range of glVRC01-class Abs *in vitro* and activate naive B cells expressing the fully humanized gl3BNC60 BCR *in vivo*. In the context of immunization, we do not presently know whether an immunogen that binds a broad range of germline glVRC01 class precursors with low affinity will be more effective in initiating an expansion of glVRC01 class B cells than an immunogen that binds a narrower range of glVRC01 class precursors but with high affinity/avidity. In a natural setting, B cells recognizing epitopes distinct from that of VRC01 may also be activated by our immunogen. B-cell competition between these B cells and glVRC01 progenitors may delay the maturation of VRC01-class antibodies. However, as we previously reported our immunogen does not stimulate B cells against the variable regions of Env (which are highly immunogenic and are targeted primarily by non-neutralizing antibody responses) and displays limited or no recognition by B cells expressing germline BCRs of non-neutralizing CD4-BS antibodies^28^. We also note that the use of multimerized gp120-based immunogens (as discussed here) will not stimulate antibody responses to the gp41 subunit of Env, which are immunogenic and for the most part are non-neutralizing^41^.

Although the B cells in mice expressing the human knocked-in gl3BNC60 BCR display features associated with anergy, these B cells respond to multivalent display of our modified antigen. We expect that in the human circulating naive BCR repertoire^17^, there may be naive VRC01-class B cells may not recognize our optimized immunogen. It would be useful to isolate and structurally characterize such putative glVRC01-class variants to further optimize immunogen-design efforts. Furthermore, we do not know whether putative glVRC01-class BCRs will display autoreactive or anergic properties in humans. If this was the case, then that would be one additional reason why glVRC01-class bNAbs are not frequently generated during HIV-1 infection or elicited by vaccination.

Several HIV-1 bNAbs display autoreactive and/or polyreactive phenotypes and it was suggested that their elicitation by vaccination in humans may require the bypass of immunological tolerance checkpoints by various mechanisms^42^. Verkoczy *et al*.^43^ were able to overcome tolerance in mice expressing anergic 2F5 BCRs through a gp140 DNA prime immunization followed by a combination boost of MPER peptides conjugated to liposomes and TLR agonists. However, it is not clear whether multivalent antigen display, TLR agonists or a combination of both were required to break tolerance in that mouse model. Our study suggests that multimerized forms of our immunogens will be sufficient to activate naive glVRC01-class B cells during immunization regardless of any potential self-reactivity.

In sum, our study supports the notion that immunogens rationally designed to overcome evolutionary-selected, steric blocks on HIV-1 Env that prevent its recognition by specific germline BCRs, lead to the activation of the corresponding glBCRs *in vivo*. Furthermore, our results corroborate the proposal that a major reason for the lack of elicitation of VRC01-like bNAbs by previous Env immunogens is the inability of such proteins to activate the appropriate naive B cells upon immunization^12^,^13^. As such our study further illuminates a path to overcome the earliest block in the elicitation of VRC01-class bNAbs by immunization.

## Methods

### Cell lines

Recombinant HIV-1 Envelope and antibodies were expressed in Freestyle 293F cells (Life Technologies) that were not tested for mycoplasma contamination. TZM-bl cells were obtained from the NIH AIDS Reagent Program.

### Antibody production

Expression plasmids for the germline forms of NIH45-46, 3BNC60 and 12A21 were previously described^11^,^13^,^24^. The glVRC01 antibody HC expression plasmid was generated by mutagenizing the CDRH3 of the glNIH45-46 plasmid using the following primers 5′- GCGAGAGGAAAAAATTGTGATTATAATTGGGACTTCCAGCAC -3′ and 5′- GTGCTGGAAGTCCCAATTATAATCACAATTTTTTCCTCTCGC -3′. Sequences of the VH and entire light chains of VRC-PG19, VRC-PG20, VRC-PG04 and VRC-CH31 (ref. 12) were synthesized by IDT Technologies. The VH regions were used to replace the VH region of b12 in the pTT5-b12 HC vector (provided by Pamela Bjorkman California Institute of Technology) by using the EcoRI and NheI restriction sites. The VL DNA was synthesized by IDT and used to replace the VL in pTT3-b12 light chain^11^ or pTT3-PG9 light chain^44^ for kappa and lambda, respectively Plasmids expressing the Fab forms of these antibodies were produced by inserting a 6-His tag followed by a stop codon directly after the C1 region of the heavy chain expression plasmids (forward Fab mutagenesis primer, 5′- caaatcttgtgacaaaactcaccatcaccatcaccattgacagcacctgaactcctggggggac -3′).

sIgGs were produced by co-transfecting the appropriate heavy and light chain plasmids at a 1:1 ratio into Freestyle 293F cells at a density of 10^6^ cells ml^−1^ in Freestyle 293 media (Life Technologies) using the 293Free transfection reagent (EMD Millipore). Antibody expression was carried out in Freestyle 293 media for 6 days with gentle shaking at 37 °C in the presence of 5% CO_2_ after which cells and cellular debris were removed by centrifugation at 10,000*g* followed by filtration through a 0.2 μM filter. Supernatants were then applied to Pierce Protein A Agarose (Thermo Scientific) followed by washing with phosphate-buffered saline (PBS). Antibodies were eluted in 1 ml fractions with Pierce IgG Elution Buffer pH 2.0 (Thermo Scientific) into 1.5 ml centrifuge tubes containing 0.1 ml of 1 M Tris-HCl pH 8.0. Fractions containing protein were pooled and exchanged into PBS using Zebra spin desalting columns (Thermo Scientific).

Fab fragments were produced in a similar manner. Following filtration, the clarified supernatant was then passed over Ni-NTA resin (Qiagen, Valencia, CA), pre-equilbrated with Ni-NTA binding buffer, followed by extensive washing with Ni-NTA binding buffer supplemented with 10 mM imidazole, and then eluted with 250 mM imidazole, 0.3 M NaCl, 20 mM Tris, pH 8.0. Ni-NTA FAb fragments were further purified by SEC using a 10/300 S200 column (GE Healthcare) equilibrated in PBS.

### Recombinant envelopes

All constructs are based on the clade C 426c gp140 (GenBank: KC769518.1; ref. 13) or the 426c.NLGS.TMΔV1-3 (herein called 426c.TM1ΔV1-3) expressed from the pTT3 vector with or without a C-terminal Avi-Tag^28^ unless otherwise noted. Amino-acid substitutions (summarized in Table 1) were generated by site-directed mutagenesis using the Stratagene Quick Change II system (Agilent Technologies, Santa Clara, CA) with primers designed using Agilent's QuikChange Primer Design Program and synthesized by Integrated DNA Technologies (IDT, Coralville, Iowa). All mutations were confirmed by Sanger sequencing.

The gp160 plasmids encoding 426c.N276D.N460D.N463D, 426c.S276A.T462A.T465A, 426c.S278R.N460D.N463D, Q168a2.N462D.N465D, Q461e2.N276D.N463D, Bal.N276D.N463D, 823c.N276D.N463D and 706c.N276D.N463D were generated by introducing mutations into the parental gp160 plasmids (Genbank numbers, KC769518.1, AF407148.1, AF407156.1, DQ318210.1, KC769511.1 and KC769513.1, respectively) using Agilent's QuikChange Primer Design Program and synthesized by Integrated DNA Technologies (IDT). All mutations were confirmed by Sanger sequencing.

The cDNA for 426cTM4ΔV1-3 amino acids 44-494 (HXB2 numbering) followed by a GSGGGGSG and the previously described *Helicobacter pylori* bullfrog ferritin^35^ was codon optimized for human, synthesized by IDT technologies and cloned into the pTT3 expression vector to create pTT3-426cTM4ΔV1-3-ferritin.

cDNA for 426cTM4ΔV1-3 amino acids 44-494 was PCR amplified from pTT3-426cTM4ΔV1-3-ferritin and subcloned into the pCVL-UCOE0.7-SFFV-C4b-IRES-GFP parental vector encoding the C4b heptamerization motif:WETPEGCEQVLTGKRLMQ CLPNPEDVKMALEVYKLSLEIEQLELQRDSARQSTLDKELVPR (Genbank: 416733). The resulting fusion protein contains an SGRAHAG linker, derived from the NotI cloning site combined with sequence from the previously crystallized construct^34^, as well as a C-terminal thrombin-6X His tag.

His-tagged 426c gp120 was engineered by disrupting the 426c furin cleavage site (RNKR →RNKG) followed immediately by the addition of a 7x-polyhisitidine tag and stop codon (forward gp120-His mutagenesis primer, 5′- ggaacaagggcgctcatcatcaccaccatcaccattgataggtggggatcggagc -3′).

### Recombinant Env-expression and purification

Plasmids encoding His-tagged Env proteins were transfected into 293 F cells at a density of 10^6^ cells ml^−1^ in Freestyle 293 media (Life Technologies) using the 293Free transfection reagent (EMD Millipore) according to the manufacturer's instructions. Expression was carried out in Freestyle 293 media for 6 days with gentle shaking at 37 °C in the presence of 5% CO_2_ after which cells and cellular debris were removed by centrifugation at 10,000*g* followed by filtration through a 0.2 μM filter. Clarified cell supernatant was passed over Ni-NTA resin (Qiagen), pre-equilbrated with Ni-NTA binding buffer (containing 5 mM imidizole), followed by extensive washing with Ni-NTA binding buffer (supplemented with 10 mM imidazole), and then eluted with 250 mM imidazole, 0.3 M NaCl, 20 mM Tris, pH 8.0. Purified gp120 proteins were then buffer exchanged into PBS using Zebra desalting columns (Thermo Scientific). Soluble trimeric gp140 Envs were expressed in 293 F cells and purified as previously described^13^. Briefly, expression plasmids encoding Env proteins were transfected into 293 F cells at a density of 10^6^ cells ml^−1^ in Freestyle 293 media (Life Technologies) using the 293Free transfection reagent (EMD Millipore) according to the manufacturer's instructions. Expression was carried out in Freestyle 293 media for 6 days with gentle shaking at 37 °C in the presence of 5% CO_2_ after which cells and cellular debris were removed by centrifugation at 10,000*g* followed by filtration through a 0.2 μM filter. Clarified cell supernatant was passed over Agarose-bound Galanthus Nivalis Lectin (GNL) resin (Vector Laboratories), pre-equilibrated with 20 mM Tris 100 mM NaCl, 1 mM EDTA pH 7.4 (GNL binding buffer), followed by extensive washing with GNL binding buffer. Bound protein was eluted with GNL binding buffer containing 1 M methyl mannopyranoside. The eluted protein was run over 16/60 S200 size-exclusion column (SEC) pre-equilibrated in PBS. Fractions containing trimeric gp140 protein were pooled, aliquoted, frozen in liquid nitrogen and stored at −20 °C. AVI-tagged Env variants were biotinylated *in vitro* using the *In Vitro* Biotin Ligase Kit (Avidity) according to the manufacturer's instructions, followed by SEC using a 10/300S 200 column (GE Healthcare) equilibrated in PBS to remove unligated biotin and BirA enzyme.

### Env multimerization

Dextramers were formed as follows. Purified biotinylated-avi tagged 426cTM4ΔV1-3 gp120 or gp140 was mixed with a biotinylated dextran (Life Technologies, Cat # D-7142) at a 3:1 ratio (Env: biotin), with the assumption that the modified dextran had 77 biotins molecules per multimer (lot-dependent value). Streptavidin (New England Biolabs Cat# N7021S) was then added to achieve a 3:1:1 Env to streptavidin to biotin ratio.

pTT3-426cTM4ΔV1-3-ferritin was transfected into 293E cells at a density of 10^6^ cells ml^−1^ in Freestyle 293 media (Life Technologies) using the 293Free transfection reagent (EMD Millipore) and half the amount of DNA recommended by the manufacturer. Expression was carried out in 293Freestyle media for 6 days with gentle shaking at 37 °C in the presence of 5% CO_2_ after which cells and cellular debris were removed by centrifugation at 10,000*g* followed by filtration through a 0.2 μM filter. Clarified supernatant was passed over a GNL agarose column (Vector Laboratories) pre-equilibrated in GNL binding buffer (20 mM Tris, 100 mM NaCl, 1 mM EDTA, pH 7.4) followed by extensive washing and then eluted with GNL binding buffer containing 1 M methylmannopyranoside. Ferritin nanoparticles were further purified by SEC using a 16/60 S200 column (GE Healthcare) equilibrated in PBS, followed by a second final SEC purification step on a 10/300 superose 6 column equilibrated in PBS (GE Healthcare).

426cTM4ΔV1-3-C4b was expressed in 293 Freestyle cells (Invitrogen) using the Daedalus system. Briefly, recombinant lentivirus was produced by transient co-transfection of 293 T using 25-kDa PEI with pCVL-UCOE0.7-SFFV-426cTM4ΔV1-3-C4b-IRES-GFP, and psPAX2 (Addgene#12260) and pMD2.G (Addgene#12259) packaging vectors. Transduction was carried out in 150 ml flasks containing 1 × 10^7^ cells in 10 ml of expression media, then cultures were expanded to a 4 l terminal culture volume. Conditioned medium was harvested by centrifugation and recombinant protein was purified using Ni-NTA.

### Biolayer interferometry

BLI assays were performed on the Octet QKe or the Octet Red instrument (ForteBio, Inc, Menlo Park, CA) at 30 °C with shaking at 1,000 r.p.m. All measurements of Env-Ab binding were corrected by subtracting the signal obtained from simultaneous traces performed with the corresponding envelopes in the absence of antibody, using PBS only. Experiments to detect glAb-binding to Env were performed as follows: Initial screening for Ab-binding was determined by immobilizing His-tagged gp120 onto Ni-NTA biosensors (ForteBio) for 300 s, sensors were then incubated with BSA (1 mg ml^−1^ in PBS) for 1 min, and the baseline signal (nm shift) was recorded for 1 min in kinetic buffer (KB: 1X PBS, 0.01% BSA, 0.02% Tween 20 and 0.005% NaN_3_). The sensors were then immersed into solutions of antibody (20 ug ml^−1^ in PBS) for 300 s, followed by immersion in KB for 300 s.

Kinetic analysis with FAbs was performed as follows: gp140s were biotinylated using EZ-Link NHS-PEG4-Biotin (Thermo Scientific) at a ratio of one biotin molecule per gp140 trimer (0.33 biotin molecules per monomer). Unligated biotin was removed using Zebra desalting columns (Thermo Scientific) according to the manufacturer's instructions. Biotinylated trimeric recombinant gp140s were immobilized on streptavidin biosensors (ForteBio) at concentrations that yielded the same *R*_max_ for all Envs tested (1–2 μM). The baseline signal was recorded for 1 min in KB, then the sensors were immersed into wells containing dilutions of purified recombinant FAbs (1–32 μM) for 4 min (association phase). The sensors were then immersed in KB without Env for an additional 8 min (dissociation phase). Curve fitting was performed using a 1:1 binding model and the Data analysis software (ForteBio). Mean *k*_on_ and *k*_off_ and *K*_A_ values were determined by averaging all binding curves that matched the theoretical fit with an *R*^2^ value of ⩾0.95.

### Generation and characterization of gl3BNC60 KI mice

The VJ_L_ sequence of gl3BNC60 mice (Genbank: KU204949) were generated in a similar way using the sequence of the predicted germline version of human 3BNC60 (ref. 11). Briefly, a targeting vector containing negative (DTA) and positive (ampicillin and kanamycin resistance) selection markers was used. The vector was further designed to encode homologous DNA regions flanking the J segments of the endogenous mouse kappa locus. Homologous recombination will therefore result in the deletion of endogenous J segments, which minimizes rearrangement of the WT locus. Chimera mice were generated by transfecting ES cells with the targeting vector. ES cells with a correctly targeted Igκ locus were then microinjected into mouse blastocysts and the resulting blastocysts were transferred into pseudopregnant recipient female mice. The pups born were screened for the knock-in gene, and LC knock-in mice were then bred to homozygosity. They were then crossed with gl3BNC60 HC (Genbank: KU204946) knock-in mice^18^,^45^,^46^ to generate double gl3BNC60 HC and LC heterozygous and eventually double homozygous mice. The knock-in HC and LC genotype was verified by PCR using specific primers for the 3BNC60 germline HC or LC sequences as well as primers specific for the WT untargeted loci of IgH and IgK. Naive B-cell development of the newly generated knock-in mice was characterized by flow cytometry. A single-cell suspension of BM was stained to identify immature (IgM^−/low^, IgD^−^) and mature (IgM^+/int^, IgD^+^) B-cell populations. Single-cell suspensions of splenocytes were stained to identify marginal zone B cells (CD23-CD21+) and follicular B cells (CD23^+^, CD21^lo/−^) and to determine kappa and lambda usage of total B cells. The following antibodies were used; anti-mouse CD4 PE-CF594, anti-mouse CD8 PE-CF594, anti-mouse Ly-6G and Ly-6C PE-CF594, anti-mouse Ig kappa BV421 (BD Biosciences), anti-Hu/Mu B220 APC-eFlour 780, anti-mouse CD19 Pe-Cy7, anti-mouse IgM PerCP-eFluor 710, anti-mouse CD21/CD35 eFluor 450 (eBiosciences), anti-mouse Ig lambda PE anti-mouse IgD Pacific Blue and anti-mouse CD23 PE (BioLegend). All the antibodies were used at a dilution of 1:200 except anti-mouse IgG1 BV421, which was used at 1:400. Live dead aqua stain was used at a dilution of 1:400 to identify dead cells (Life Technologies).

### B-cell sorting

Memory B cells of immunized knock-in mice were single-cell sorted as previously described^18^,^47^. In brief, splenocytes were stained with anti-mouse CD4 PE-CF594, anti-mouse CD8 PE-CF594, anti-mouse Ly-6G and Ly-6C (Gr1) PE-CF594, anti-mouse IgG1 BV421 (BD biosciences), anti-Hu/Mo B220 FITC, anti-mouse CD38 A700, anti-mouse IgM PerCP-eFluor 710 (eBiosciences) PE-streptavidin conjugated 426c.TM4ΔV1-3 gp140-biotin and APC-streptavidin conjugated 426c.TM4ΔV1-3.D368R.E370A-biotin (426c.TM4ΔV1-3 gp140-KO). All the antibodies were used at a dilution of 1:200 except anti-mouse IgG1 BV421, which was used at 1:400. Live dead aqua stain was used at a dilution of 1:400 to identify dead cells (Life Technologies). The sorted cells were live cells, CD4-, CD8-, Gr-1-, B220+, CD38+, IgM−, IgG1+, 426c.TM4ΔV1-3 gp140^+^, 426c.TM4ΔV1-3 gp140-KO^−^. Single cells were lysed in 4 μl of 0.5 × PBS (Nalgene), 10 mM DTT (Invitrogen) and 12 U RNAsin Plus (Promega). cDNA was synthesized by adding 7 μl of the following mix to each well; random hexamers (Invitrogen) at a concentration of 21.5 ng μl^−1^, 0.7% NP40 (Sigma) and 6 U per well of RNAsin Plus (Promega). The plates were incubated at 65 °C for 3 min and then put on ice. cDNA generation was finalized using Superscript III Reverse Transcriptase (Invitrogen) following the manufacturer's instructions. The VDJ knock-in sequence was amplified from cDNA by nested PCR using the following primer pairs; 1_3BNC60_F_HK 5′- GGGATGGTCATGTATCATCCTTTTTCTAG -3′ with 1mRG 5′- AGAAGGTGTGCACACCGCTGGAC -3′ and 2_3BNC60_F_HK 5′- GTAGCAACTGCAACCGGTGTACATTCT -3′ with 2mRG: 5′- GCTCAGGGAARTAGCCCTTGAC -3′. The VJ knock-in sequence was amplified from cDNA by nested PCR using the following primer pairs; 1_3BNC60_F_HK: 5′- GGGATGGTCATGTATCATCCTTTTTCTAG -3′ with 1mRK: 5′- ACTGAGGCACCTCCAGATGTT -3′ and 2_3BNC60_F_HK: 5′- GTAGCAACTGCAACCGGTGTACATTCT -3′ with 2mRK: 5′- TGGGAAGATGGATACAGTT -3′. 2mRG and 2mRK were used to sequence the heavy- and the light-chain products, respectively.

### Immunizations

Six-to-eight-week-old female or male WT and gl3BNC60 knock-in mice were immunized with 10 μg of recombinant Env. Three to five mice per group were used in each experiment to ensure reproducibility. Immunizations of 426c gp140, gp120-dextramer and gp140-dextramer were performed with Imject Alum (Thermo Scientific). Immunizations with gp120-dextramer were also performed with Ribi (Sigma Adjuvant System, Sigma). Immunizations with gp120-C4b were performed with Ribi. The serum was collected for analysis at 2 weeks after immunization. No randomization or blinding of the experiments was performed. All the experiments were performed according to the protocols approved by the IACUC at Rockefeller University. In the case of immunizations with dextrameric Env, the dextrameric complexes were formed for 10–15 min before the addition of Alum or Ribi. All the experiments were performed according to the protocols approved by the IACUC at Rockefeller University

### Serum ELISA

High-binding 96-well plates (Corning Incorporated) were coated with 200 ng per well of 426c.TM4 ΔV1-3 and 426c.TM4 ΔV1-3 CD4-BS KO (Corning Incorporated) with 200 ng per well of protein. After incubation overnight at 4 °C, the plates were washed in wash buffer (3 × in PBS with 0.05% TWEEN 20 (Sigma)) and blocked in blocking buffer (PBS with 2% milk). The serum samples were added to coated wells at the indicated dilutions and incubated for 1 h at 37 °C. The plates were washed and secondary antibody, HRP conjugated anti-mouse (Jackson Immuno Research), was added and incubated for 30 min at 37 °C. The plates were washed again and then developed by adding ABTS solution (Life Technologies) and the absorbance was measured at 405 nm using a FLUOstar Omega microplate reader (BMG Labtech) which gives a maxiumum reading of 4.0.

### MAb ELISA

A total 100 μl per well of HIV Env proteins (0.5 μg ml^−1^ in 0.1 M NaHCO_3_, pH 9.5) were adsorbed onto 96-well ELISA plates (Immulon 2HB) overnight at room temperature. Wells were washed four times in PBS+0.02% Tween 20 (ELISA wash buffer) and then blocked with 300 μl per well of PBS containing 10% milk 0.03% Tween 20 for 2 h at 37 °C followed by four washes with ELISA wash buffer. Antibodies were diluted in PBS containing 10% milk; 0.03% Tween 20 (incubation buffer) were added to the wells and incubated for 1 h at 37 °C and then washed four times in ELISA wash buffer. HRP-conjugated goat anti-human IgG secondary antibody (Southern BioTech, Birmingham, AL) was added to the wells (1:3,000 in dilution buffer) and incubated at 37 °C for 1 h and then washed four times in ELISA wash buffer. Then, 50 μl of Ultra TMB substrate (Thermo Scientific) was added to each well. After 3 min at room temperature, an equal volume of 1 N H_2_SO_4_ was added. The absorbance at 450 nM was measured using a Spectramax M2 plate reader.

### Capture ELISA

Plasmids expressing 426c.N276D.N460D.N463D, Q168a2.N462D.N465D, Q461e2.N276D.N463D, Bal.N276D.N463D, 823c.N276D.N463D and 706c.N276D.N463D gp160 proteins were transfected into 293 cells using GeneJuice (Merck Millipore) according to the manufacturer's instructions. Seventy-two hours later, the cells were lysed with PBS containing 1% Triton X-100. Cell lysates were clarified by centrifugation, passed through a 0.2 μM filter and then incubated on ELISA plates coated with the anti-C-terminal D7324 anti-gp120 sheep antibody (Aalto Bioreagents) for 1 h at 37 °C. Wells were washed four times in ELISA wash buffer and then blocked with 300 μl per well of PBS containing 10% milk and 0.03% Tween 20 for 2 h at 37 °C followed by four washes with ELISA wash buffer.

NIH45-46 and VRC01 antibodies were diluted to 15 μg ml^−1^ (germline) and 2 μg ml^−1^ (mature) in incubation buffer. Then, 100 μl per well of each antibody dilution was added to plates containing captured cell lysates in triplicate, and incubated for 1 h at 37 °C. The plates were then washed four times in ELISA wash buffer. HRP-conjugated goat anti-human IgG secondary antibody (Southern BioTech, Birmingham, AL) was added to the wells (1:3,000 in dilution buffer) and incubated at 37 °C for 1 h and then washed four times in ELISA wash buffer. Then, 50 μl of Ultra TMB substrate (Thermo Scientific) was added to each well. After 3 min at room temperature, an equal volume of 1 N H_2_SO_4_ was added. The absorbance at 450 nM was measured using a Spectramax M2 plate reader.

### Neutralization assay

Serum IgG were tested against a panel of HIV-1 pseudoviruses using the TZM-bl neutralization assay as previously described^48^. Briefly, 200 TCID_50_ of virus was incubated with various dilutions of test samples (eight dilutions, threefold stepwise) in duplicate for 1 h at 37 °C in a total volume of 150 μl growth medium in 96-well flat-bottom culture plates (Corning-Costar). Freshly trypsinized TZM-bl cells (10,000 cells in 100 μl of growth medium containing 75 μg ml^−1^ DEAE-dextran) were added to each well. One set of eight control wells received cells plus virus (virus control), and another set of eight wells received cells only (background control). After a 48-h incubation, 150 μl of culture medium was removed from each well and 100 μl of Bright Glo reagent was added to the cells. After a 2-min incubation at room temperature to allow cell lysis, 150 μl of cell lysate was transferred to 96-well black solid plates for measurements of luminescence using a Victor 2 luminometer. The 50% inhibitory dose (ID_50_) was defined as the serum that caused a 50% reduction in relative luciferase units (RLU) compared with virus control wells after subtraction of background RLU.

## Additional information

**Accession codes:** The isolated heavy-chain (KU204946-KU204948) and light-chain (KU204949-KU204954) sequences have been deposited in Genbank.

**How to cite this article**: McGuire, A. T. *et al*. Specifically modified Env immunogens activate B-cell precursors of broadly neutralizing HIV-1 antibodies in transgenic mice. *Nat. Commun.* 7:10618 doi: 10.1038/ncomms10618 (2016).

## Supplementary Material

Supplementary Information Supplementary Figures 1-6 and Supplementary Tables 1-3

## Figures and Tables

**Table 1:** Summary of the various mutations introduced onto the 426c Env background.

**Envelope**	**WT**	**N460D N463D**	**TM1**	**TM2**	**TM3**	**TM4**	**TM5**	**TM1**Δ**V1**–**3**	**TM4**Δ**V1**–**3**	**TM5**Δ**V1**–**3**
**Mutations**	None	N460DN463D	N276DN460DN463D	S278AN460DN463D	S278RN460DN463D	S278RN460DN463DG471S	N276DS278RN460DN463DG471S	N276DN460DN463DΔV1 V2 V3	S278RN460DN463DG471SΔV1 V2 V3	N276DS278RN460DN463DG471SΔV1 V2 V3

## References

[b1] Burton D. R. & Mascola J. R. Antibody responses to envelope glycoproteins in HIV-1 infection. Nat. Immunol. 16, 571–576 (2015). 25988889 10.1038/ni.3158 PMC4834917

[b2] West A. P. Jr . Structural insights on the role of antibodies in HIV-1 vaccine and therapy. Cell 156, 633–648 (2014). 24529371 10.1016/j.cell.2014.01.052 PMC4041625

[b3] Zhou T. . Structural repertoire of HIV-1-neutralizing antibodies targeting the CD4 supersite in 14 donors. Cell 161, 1280–1292 (2015). 26004070 10.1016/j.cell.2015.05.007 PMC4683157

[b4] Rudicell R. S. . Enhanced potency of a broadly neutralizing HIV-1 antibody *in vitro* improves protection against lentiviral infection *in vivo*. J. Virol. 88, 12669–12682 (2014). 25142607 10.1128/JVI.02213-14 PMC4248941

[b5] Shingai M. . Passive transfer of modest titers of potent and broadly neutralizing anti-HIV monoclonal antibodies block SHIV infection in macaques. J. Exp. Med. 211, 2061–2074 (2014). 25155019 10.1084/jem.20132494 PMC4172223

[b6] Saunders K. O. . Sustained delivery of a broadly neutralizing antibody in nonhuman primates confers long-term protection against simian/human immunodeficiency virus infection. J. Virol. 89, 5895–5903 (2015). 25787288 10.1128/JVI.00210-15 PMC4442454

[b7] Saunders K. O. . Broadly neutralizing human immunodeficiency virus type 1 antibody gene transfer protects nonhuman primates from mucosal simian-human immunodeficiency virus infection. J. Virol. 89, 8334–8345 (2015). 26041300 10.1128/JVI.00908-15 PMC4524228

[b8] Deal C. E. & Balazs A. B. Vectored antibody gene delivery for the prevention or treatment of HIV infection. Curr. Opin. HIV AIDS 10, 190–197 (2015). 25700206 10.1097/COH.0000000000000145 PMC4448693

[b9] Gruell H. . Antibody and antiretroviral preexposure prophylaxis prevent cervicovaginal HIV-1 infection in a transgenic mouse model. J. Virol. 87, 8535–8544 (2013). 23720722 10.1128/JVI.00868-13 PMC3719827

[b10] McCoy L. E. . Potent and broad neutralization of HIV-1 by a llama antibody elicited by immunization. J. Exp. Med. 209, 1091–1103 (2012). 22641382 10.1084/jem.20112655 PMC3371729

[b11] Hoot S. . Recombinant HIV envelope proteins fail to engage germline versions of anti-CD4bs bNAbs. PLoS Pathog. 9, e1003106 (2013). 23300456 10.1371/journal.ppat.1003106 PMC3536657

[b12] Jardine J. . Rational HIV immunogen design to target specific germline B cell receptors. Science 340, 711–716 (2013). 23539181 10.1126/science.1234150 PMC3689846

[b13] McGuire A. T. . Engineering HIV envelope protein to activate germline B cell receptors of broadly neutralizing anti-CD4 binding site antibodies. J. Exp. Med. 210, 655–663 (2013). 23530120 10.1084/jem.20122824 PMC3620356

[b14] Diskin R. . Increasing the potency and breadth of an HIV antibody by using structure-based rational design. Science 334, 1289–1293 (2011). 22033520 10.1126/science.1213782 PMC3232316

[b15] Zhou T. . Structural basis for broad and potent neutralization of HIV-1 by antibody VRC01. Science 329, 811–817 (2010). 20616231 10.1126/science.1192819 PMC2981354

[b16] Zhou T. . Multidonor analysis reveals structural elements, genetic determinants, and maturation pathway for HIV-1 neutralization by VRC01-class antibodies. Immunity 39, 245–258 (2013). 23911655 10.1016/j.immuni.2013.04.012 PMC3985390

[b17] DeKosky B. J. . In-depth determination and analysis of the human paired heavy- and light-chain antibody repertoire. Nat. Med. 21, 86–91 (2015). 25501908 10.1038/nm.3743

[b18] Dosenovic P. . Immunization for HIV-1 broadly neutralizing antibodies in human Ig knockin mice. Cell 161, 1505–1515 (2015). 26091035 10.1016/j.cell.2015.06.003 PMC4604566

[b19] Finton K. A. . Autoreactivity and exceptional CDR plasticity (but not unusual polyspecificity) hinder elicitation of the anti-HIV antibody 4E10. PLoS Pathog. 9, e1003639 (2013). 24086134 10.1371/journal.ppat.1003639 PMC3784475

[b20] Chen Y. . Common tolerance mechanisms, but distinct cross-reactivities associated with gp41 and lipids, limit production of HIV-1 broad neutralizing antibodies 2F5 and 4E10. J. Immunol. 191, 1260–1275 (2013). 23825311 10.4049/jimmunol.1300770 PMC3725147

[b21] Doyle-Cooper C. . Immune tolerance negatively regulates B cells in knock-in mice expressing broadly neutralizing HIV antibody 4E10. J. Immunol. 191, 3186–3191 (2013). 23940276 10.4049/jimmunol.1301285 PMC3773228

[b22] Nemazee D. A. & Burki K. Clonal deletion of B lymphocytes in a transgenic mouse bearing anti-MHC class I antibody genes. Nature 337, 562–566 (1989). 2783762 10.1038/337562a0

[b23] Goodnow C. C. . Altered immunoglobulin expression and functional silencing of self-reactive B lymphocytes in transgenic mice. Nature 334, 676–682 (1988). 3261841 10.1038/334676a0

[b24] Scheid J. F. . Sequence and structural convergence of broad and potent HIV antibodies that mimic CD4 binding. Science 333, 1633–1637 (2011). 21764753 10.1126/science.1207227 PMC3351836

[b25] Wu X. . Focused evolution of HIV-1 neutralizing antibodies revealed by structures and deep sequencing. Science 333, 1593–1602 (2011). 21835983 10.1126/science.1207532 PMC3516815

[b26] Wu X. . Maturation and diversity of the VRC01-antibody lineage over 15 years of chronic HIV-1 infection. Cell 161, 470–485 (2015). 25865483 10.1016/j.cell.2015.03.004 PMC4706178

[b27] Georgiev I. S. . Delineating antibody recognition in polyclonal sera from patterns of HIV-1 isolate neutralization. Science 340, 751–756 (2013). 23661761 10.1126/science.1233989

[b28] McGuire A. T. . HIV antibodies. Antigen modification regulates competition of broad and narrow neutralizing HIV antibodies. Science 346, 1380–1383 (2014). 25504724 10.1126/science.1259206 PMC4290850

[b29] West A. P. Jr., Diskin R., Nussenzweig M. C. & Bjorkman P. J. Structural basis for germ-line gene usage of a potent class of antibodies targeting the CD4-binding site of HIV-1 gp120. Proc. Natl Acad. Sci. USA 109, E2083–E2090 (2012). 22745174 10.1073/pnas.1208984109 PMC3409792

[b30] Tran K. . Vaccine-elicited primate antibodies use a distinct approach to the HIV-1 primary receptor binding site informing vaccine redesign. Proc. Natl Acad. Sci. USA 111, E738–E747 (2014). 24550318 10.1073/pnas.1319512111 PMC3932900

[b31] Cooke M. P. . Immunoglobulin signal transduction guides the specificity of B cell-T cell interactions and is blocked in tolerant self-reactive B cells. J. Exp. Med. 179, 425–438 (1994). 8294858 10.1084/jem.179.2.425 PMC2191355

[b32] Batista F. D. & Neuberger M. S. Affinity dependence of the B cell response to antigen: a threshold, a ceiling, and the importance of off-rate. Immunity 8, 751–759 (1998). 9655489 10.1016/s1074-7613(00)80580-4

[b33] Sabouri Z. . Redemption of autoantibodies on anergic B cells by variable-region glycosylation and mutation away from self-reactivity. Proc. Natl Acad. Sci. USA 111, E2567–E2575 (2014). 24821781 10.1073/pnas.1406974111 PMC4078846

[b34] Hofmeyer T. . Arranged sevenfold: structural insights into the C-terminal oligomerization domain of human C4b-binding protein. J. Mol. Biol. 425, 1302–1317 (2013). 23274142 10.1016/j.jmb.2012.12.017

[b35] Kanekiyo M. . Rational design of an Epstein-Barr virus vaccine targeting the receptor-binding site. Cell 162, 1090–1100 (2015). 26279189 10.1016/j.cell.2015.07.043 PMC4757492

[b36] Jardine J. G. . HIV-1 vaccines. Priming a broadly neutralizing antibody response to HIV-1 using a germline-targeting immunogen. Science 349, 156–161 (2015). 26089355 10.1126/science.aac5894 PMC4669217

[b37] Xiao X., Chen W., Feng Y. & Dimitrov D. S. Maturation pathways of cross-reactive HIV-1 neutralizing antibodies. Viruses 1, 802–817 (2009). 21994570 10.3390/v1030802 PMC3185542

[b38] Klein F. . Antibodies in HIV-1 vaccine development and therapy. Science 341, 1199–1204 (2013). 24031012 10.1126/science.1241144 PMC3970325

[b39] Doria-Rose N. A. . Developmental pathway for potent V1V2-directed HIV-neutralizing antibodies. Nature 509, 55–62 (2014). 24590074 10.1038/nature13036 PMC4395007

[b40] Liao H. X. . Co-evolution of a broadly neutralizing HIV-1 antibody and founder virus. Nature 496, 469–476 (2013). 23552890 10.1038/nature12053 PMC3637846

[b41] Williams W. B. . HIV-1 VACCINES. Diversion of HIV-1 vaccine-induced immunity by gp41-microbiota cross-reactive antibodies. Science 349, aab1253 (2015). 26229114 10.1126/science.aab1253 PMC4562404

[b42] Liu M. . Polyreactivity and autoreactivity among HIV-1 antibodies. J. Virol. 89, 784–798 (2015). 25355869 10.1128/JVI.02378-14 PMC4301171

[b43] Verkoczy L. . Induction of HIV-1 broad neutralizing antibodies in 2F5 knock-in mice: selection against membrane proximal external region-associated autoreactivity limits T-dependent responses. J. Immunol. 191, 2538–2550 (2013). 23918977 10.4049/jimmunol.1300971 PMC3870053

[b44] McGuire A. T., Glenn J. A., Lippy A. & Stamatatos L. Diverse recombinant HIV-1 Envs fail to activate B cells expressing the germline B cell receptors of the broadly neutralizing anti-HIV-1 antibodies PG9 and 447-52D. J. Virol. 88, 2645–2657 (2013). 24352455 10.1128/JVI.03228-13 PMC3958080

[b45] Pelanda R. . Receptor editing in a transgenic mouse model: site, efficiency, and role in B cell tolerance and antibody diversification. Immunity 7, 765–775 (1997). 9430222 10.1016/s1074-7613(00)80395-7

[b46] Shih T. A., Roederer M. & Nussenzweig M. C. Role of antigen receptor affinity in T cell-independent antibody responses *in vivo*. Nat. Immunol. 3, 399–406 (2002). 11896394 10.1038/ni776

[b47] Tiller T., Busse C. E. & Wardemann H. Cloning and expression of murine Ig genes from single B cells. J. Immunol. Methods 350, 183–193 (2009). 19716372 10.1016/j.jim.2009.08.009

[b48] Li M. . Human immunodeficiency virus type 1 env clones from acute and early subtype B infections for standardized assessments of vaccine-elicited neutralizing antibodies. J. Virol. 79, 10108–10125 (2005). 16051804 10.1128/JVI.79.16.10108-10125.2005 PMC1182643

