# Supplementary material for: Specifically modified Env immunogens activate B-cell precursors of broadly neutralizing HIV-1 antibodies in transgenic mice
Source: Nat Commun. 2016 Feb 24;7:10618. doi: 10.1038/ncomms10618 (PMC4770077; doi:10.1038/ncomms10618)
Supplement: Supplementary Information — Supplementary Figures 1-6 and Supplementary Tables 1-3 [file ncomms10618-s1.pdf]

```
                                           CDRL1                       CDRL2                                 CDRL3
glVRC/NIH   EIVLTQSPATLSLSPGERATLSCR---ASQSVSSYLAWYQQKPGQAPRLLIYDASNRATGIPARFSGSGSGTDFTLTISSLEPEDFAVYYCQQYEFFGQGTKLEIK
gl12A21     DIQMTQSPSSLSASVGDRVTITCQ---ASQDISNYLNWYQQKPGKAPKLLIYDASNLETGVPSRFSGSGSGTDFTFTISSLQPEDIATYYCAVLEFFGPGTKVDIK
gl3BNC60    DIQMTQSPSSLSASVGDRVTITCQ---ASQDISNYLNWYQQKPGKAPKLLIYDASNLETGVPSRFSGSGSGTDFTFTISSLQPEDIATYYCQQYEFIGPGTKVDIK
glVRC-CH31  DIQMTQSPSSLSASVGDRVTITCQ---ASQDISNYLNWYQQKPGKAPKLLIYDASNLETGVPSRFSGSGSGTDFTFTISSLQPEDIATYYCQQYETFGQGTKLEIK
glPGV04     EIVLTQSPGTLSLSPGERATLSCR--ASQSVSSSYLAWYQQKPGQAPRLLIYGASSRATGIPDRFSGSGSGTDFTLTISRLEPEDFAVYYCQQLEFFGQGTRLEIK
glPGV19/20  -QSALTQPASVSGSPGQSITISCTGTSSDVGGYNYVSWYQQHPGKAPKLMIYEVSNRPSGVSNRFSGSKSGNTASLTISGLQAEDEADYYCSSYEFFGGGTKVFVL
```

Supplementary Figure 1. Amino acid alignment of the light chain variable regions of germline VRC01-class antibodies. Gray areas indicate (Kabat) CDRL regions. Blue letters indicate negatively charged amino acids and red letters indicate positively charged amino acids.

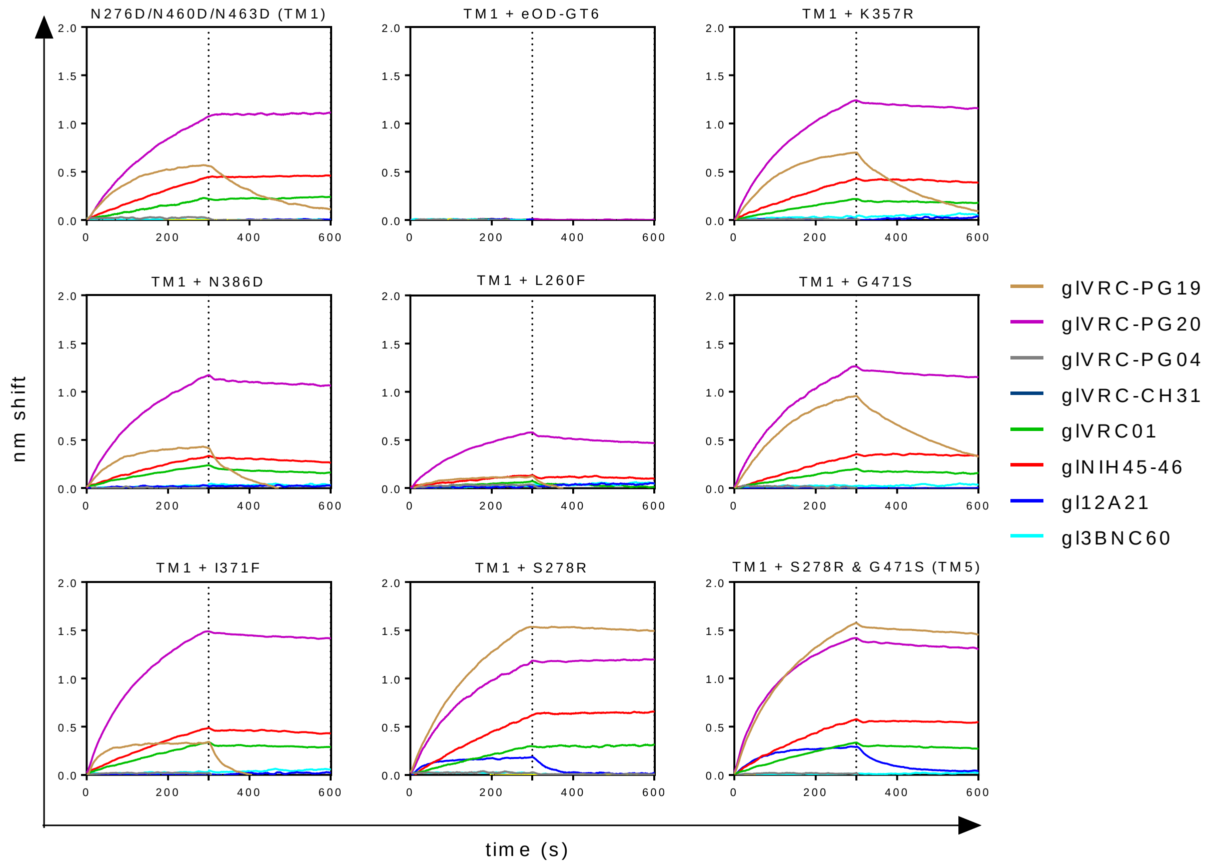

Supplementary Figure 2. Binding of glVRC01-class antibodies to 426c Env with mutations previously identified as improving antibody-recognition of the outer domain of gp120, eOD-GT6. The interaction of the indicated gl antibodies to gp120 variants of 426cTM1 with six additional mutations identified to improve glVRC01-class antibodies to eOD-GT6[12] were determined by BLI. BLI traces are representative of at least 3 independent replicates.

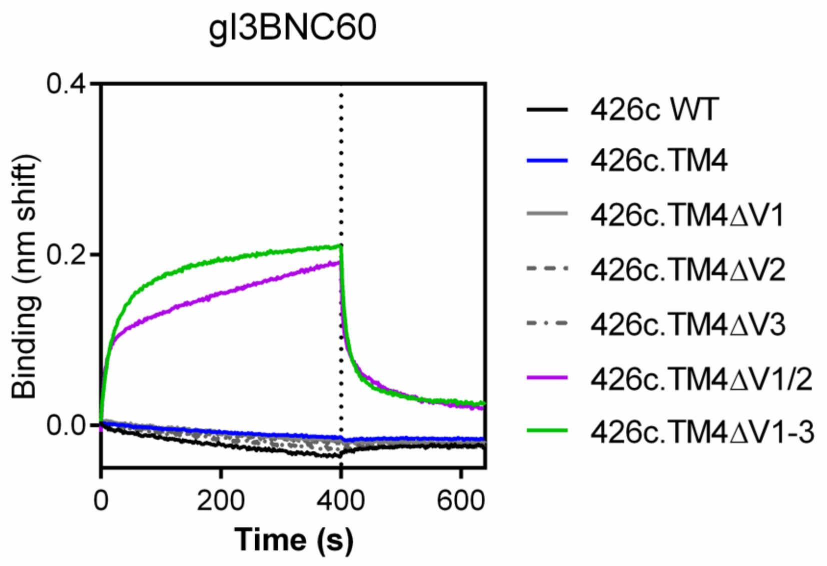

Supplementary Figure 3. Effect of Env variable loop deletions on the binding of gl3BNC60. Binding of gl3BNC60 to 426cTM4 gp120, or to 426cTM4 gp120 with the variable regions 1, 2 and 3 deleted individually (426cTM4ΔV1, 426cTM4ΔV2 and 426cTM4ΔV3, respectively), or in combination (426cTM4ΔV1/2 and 426cTM4ΔV1-3). BLI traces are representative of 2 independent replicates.

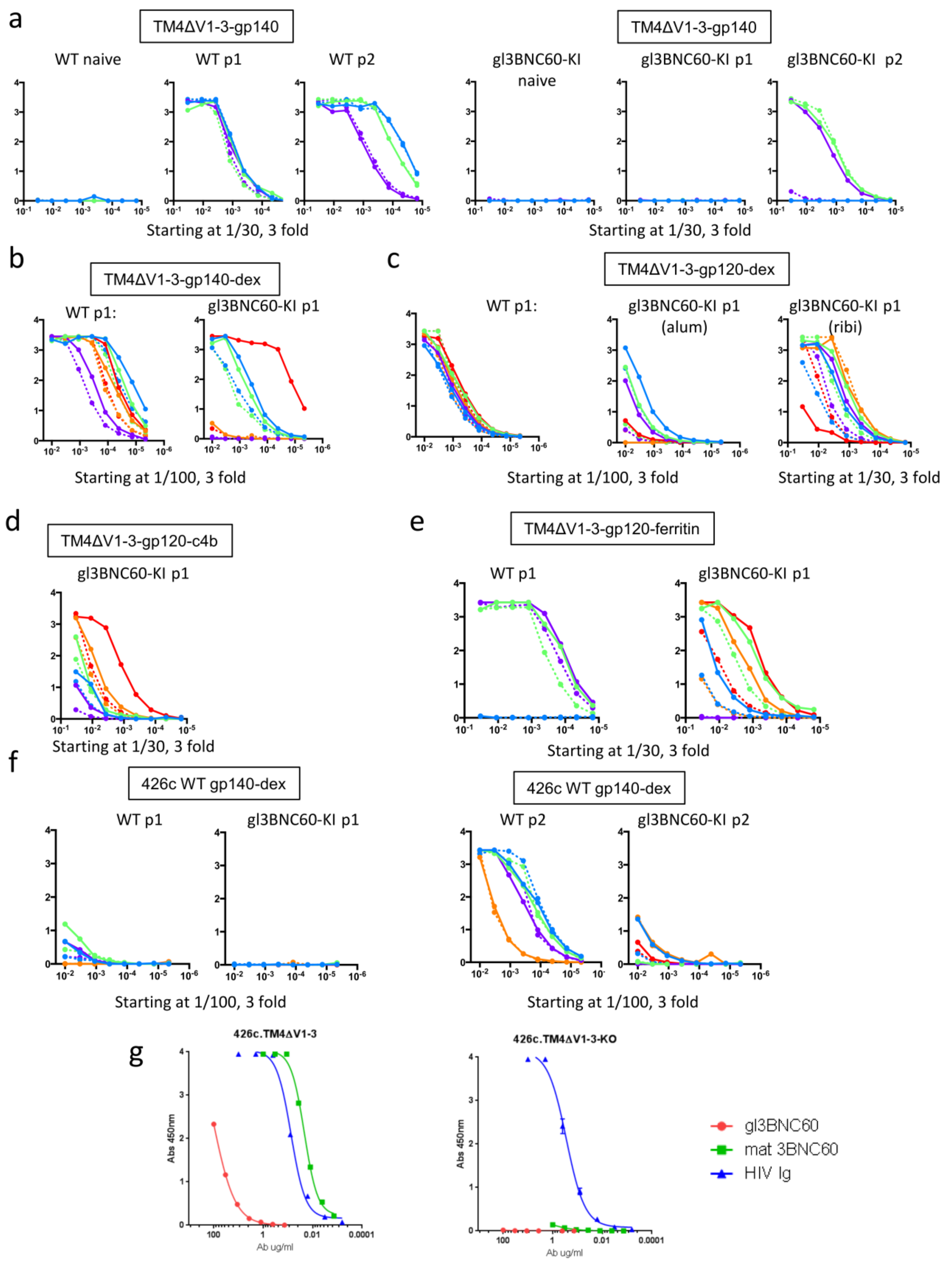
a
TM4ΔV1-3-gp140
WT naive
WT p1
WT p2
Starting at 1/30, 3 fold
TM4ΔV1-3-gp140
gl3BNC60-KI naive
gl3BNC60-KI p1
gl3BNC60-KI p2
Starting at 1/30, 3 fold
b
TM4ΔV1-3-gp140-dex
WT p1:
gl3BNC60-KI p1
Starting at 1/100, 3 fold
c
TM4ΔV1-3-gp120-dex
WT p1:
gl3BNC60-KI p1 (alum)
Starting at 1/100, 3 fold
gl3BNC60-KI p1 (ribi)
Starting at 1/30, 3 fold
d
TM4ΔV1-3-gp120-c4b
gl3BNC60-KI p1
Starting at 1/30, 3 fold
e
TM4ΔV1-3-gp120-ferritin
WT p1
gl3BNC60-KI p1
Starting at 1/30, 3 fold
f
426c WT gp140-dex
WT p1
gl3BNC60-KI p1
Starting at 1/100, 3 fold
426c WT gp140-dex
WT p2
gl3BNC60-KI p2
Starting at 1/100, 3 fold
g
426c.TM4ΔV1-3
Abs 450nm
Ab ug/ml
426c.TM4ΔV1-3-KO
Abs 450nm
Ab ug/ml
gl3BNC60
mat 3BNC60
HIV Ig

Supplementary Figure 4. Antibody responses elicited in WT and knock-in gl3BNC60 mice after immunization.

(**a**)Serum IgG collected prior to (naïve) and following one or two immunizations (post 1, 2) with 426cTM4ΔV1-V3 gp140 in Alum Imject were tested for binding to 426cTM4ΔV1-V3 gp140 (solid lines) or 426cTM4ΔV1-V3.gp140.D368R.E370A protein (KO) (dotted lines) in WT (left three panels, n=3) and knock-in gl3BNC60 mice (right three panels, n=3) by ELISA. Colors indicate individual mice. (**b**) Same as in **a** but WT mice (n=5, left panel) or gl3BNC60 KI (n=5, right panel) mice were immunized once with 426cTM4ΔV1-V3 gp140 dextramer in Alum Imject. (**c**) Same as in **a** but WT (n=5, left panel) or gl3BNC60 KI mice (n=5, middle panel) were immunized once with 426cTM4ΔV1-V3 gp120-dextramers in Alum Imject, or in Ribi adjuvant (n=5, right panel). **(d)** Same as in **a** but gl3BNC60 KI mice (n=5) were immunized once with 426c.TM4ΔV1-V3 gp120-C4b in Ribi adjuvant. (**e**) Same as in **a** but WT mice (n=3, left panel) or gl3BNC60 KI mice (n=5, right panel) were immunized once with 426cTM4ΔV1-V3 gp120-ferritin in Alum Imject. The red and orange lines correspond to the serum responses of the mice that were used for sorting in Figs.4, S6 and Table S3. (**f**) Serum IgG from WT (n=4)and 3BNC60 KI mice (n=5) after one (p1, left two panels) or two (p2, right two panels) immunizations with WT 426c gp140-dextramer in Alum Imject were tested for binding to WT 426c gp140 (solid lines) or 426c.D368R.E370A (426c-KO, dotted lines) by ELISA. (**g**) gl3BNC60 (red curves), mut3BNC60 (green curves) and polyclonal HIV-IgG reagent (blue curves) were tested for binding to 426cTM4ΔV1-V3 (left panel) or 426cTM4ΔV1-V3-KO (right panel).

a

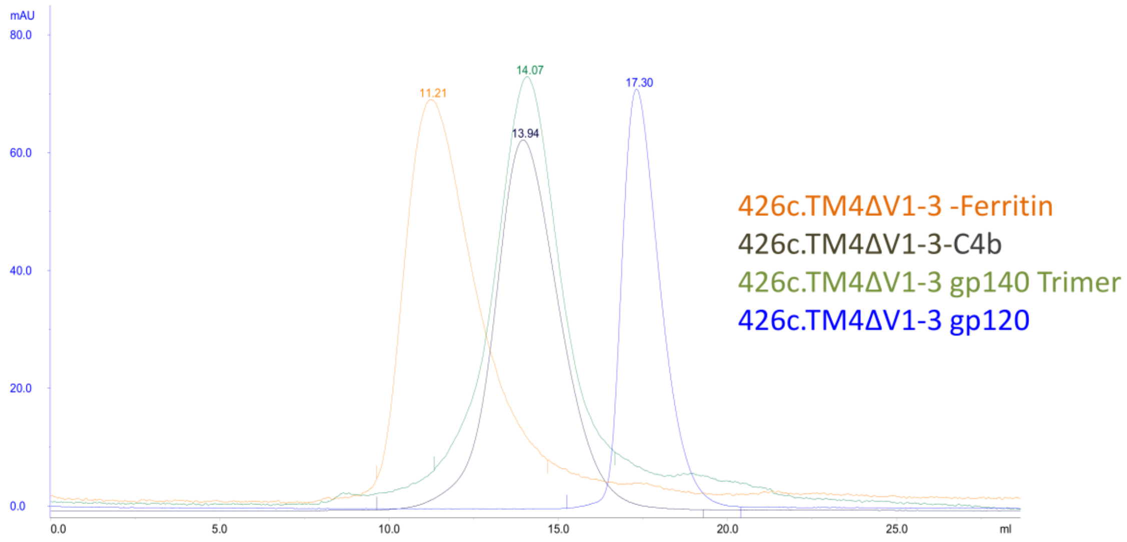

b

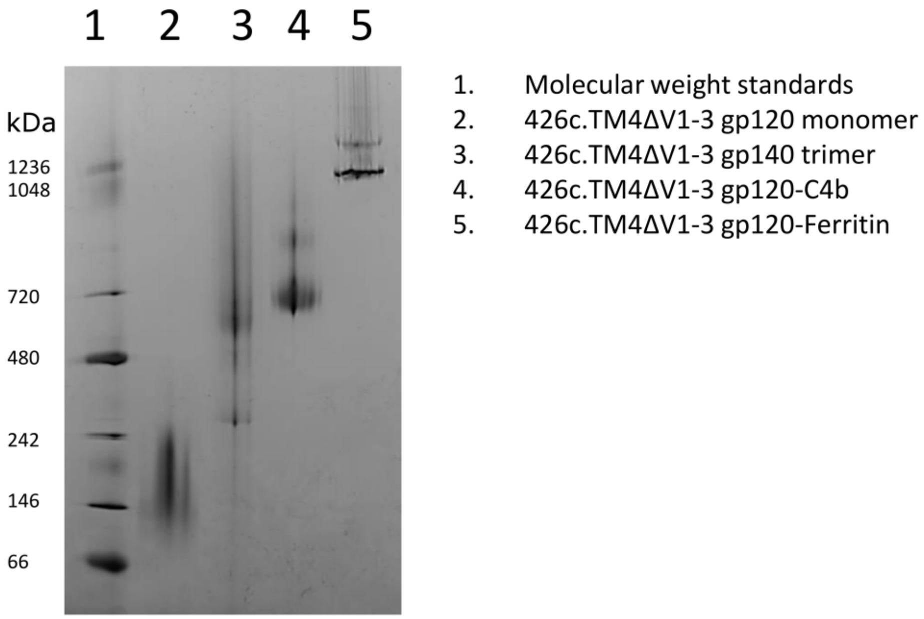

Supplementary Figure 5. Biochemical characterization of 426cTM4∆V1-3 multimers.
(**a**) Chromatograms of the indicated multimeric 426cTM4ΔV1-3 variants run on a 10/300 Superose 6 column. The elution volume is indicated above the peak of each trace. (**b**) 10 μg of the indicated multimeric 426cTM4ΔV1-3 variants were subjected to BN-PAGE.

a

	1	2	3	4	5	6	7	8	9	10	11	12
A	H		L	L	L	H	H		L			L
B		HL	HL	L	L	L		HL	L	L	L	L
C	L	L		L	L	L	L	L	L	L	H	L
D	L	L		L	L	L	HL	L	L			L
E	L		L	L	L	L	H		L	L	L	L
F		L	L	L	L	L		L	L		L	L
G	H		L	HL			L	L	L	L		L
H	X	X	X	X	X			L				HL

b

```
Heavy Chain
                                            CDRH1                   CDRH2                                                   CDRH3
gl3BNC60(KU204946)    QVQLVQSGAEVKKPGASVKVSCKASGYTFTGYYMHWVRQAPGQGLEWMGWINPNSGGTNYAQKFQGRVTMTRDTS----ISTAYMELSRLRSDDTAVYYCARERSDF--WDFDLWGRGTLVTVSS
HC B2  (KU204947)     ..........................H................................................----............................--................
HC B8  (KU204948)     .....................................Q.....................................----............................--................
3BNC60(HE584535.1)    ..H.S....A.T......R...E....KISDHFI..W........Q.V.....KT.QP.NPRQ.....SL..QA.WDFDTYSF..D.KAV......I.F...Q....--....V..S..Q.....
VRC01  (GU980702.1)   ........GQM....E.MRI..R....E.IDCTLN.I.L...KRP.....LK.RG.AV...RPL.........VY----SD..FL..RS.TV......F.T.GKNCDYN...EH.....P.I...

Light Chain
                                          CDRL1                CDRL2                                     CDRL3
gl3BNC60 (KU204949)   DIQMTQSPSSLSASVGDRVTITCQASQDISNYLNWYQQKPGKAPKLLIYDASNLETGVPSRFSGSGSGTDFTFTISSLQPEDIATYYCQQYEFIGPGTKVDIKR
LC F4   (KU204950)    ............................................................................N...........................
LC C6   (KU204952)    ...................................F...........L...........................G............................
LC E11  (KU204953)    ..................................CF.H.S.............................A.SS.........F.....................
3BNCN60(HE584536.1)   .............R...T.......----NG.......RR..........G.K..R...A....RRW.QEYNL..NN.....V...F..V....V...RL.L..
```

Supplementary Figure 6. gl3BNC60 sequences isolated from antigen-specific IgG$^+$ B cells post immunization. IgG$^+$ B cells were sorted into single wells of a 96 well plate and VH and VK regions were amplified by PCR and Sanger sequenced. (**a**) Wells where a heavy, or light chain were amplified are indicated by "**H**" and "**L**", respectively. Blank wells are indicated by an "**X**". Wells shaded with a grey background contain cells sorted from the mouse with the serum response represented by the red curve in figure S4e. White wells contain cells sorted from the mouse with the serum response represented by the orange curve in figure S4e. Red letters indicate sequences where at least one amino substitution was observed. (**b**) Of 61 light chains that were recovered, 3 had mutations that led to amino acid substitutions (bottom). Mutated light chains, as well as the 3BNC60 light chain aligned to the gl3BNC60 light chain sequence (top). Genbank accession numbers are included in parentheses.

Supplementary Table 1. Binding kinetic values for the indicated antibodies to the indicated 426c based, trimeric gp140 constructs from Figure 2.

Env Name	K_A(M-1)	k_{on} ($M^{-1}s^{-1}$)	k_{on} error	k_{off} (s^{-1})	k_{off} error
**glVRC01**					
TM1	1.31E+06	1.48E+04	7.22E+02	1.13E-02	1.83E-04
TM2	6.28E+05	3.63E+04	3.10E+03	5.79E-02	1.66E-03
TM3	5.62E+05	3.60E+04	2.12E+03	6.40E-02	1.16E-03
TM4	1.08E+06	2.87E+04	1.07E+03	2.64E-02	3.21E-04
TM5	1.14E+07	2.56E+04	2.26E+02	2.25E-03	7.24E-06
TM1ΔV1-3	3.09E+06	4.84E+03	3.34E+01	1.57E-03	5.29E-06
TM4ΔV1-3	1.47E+06	6.25E+03	7.05E+01	4.26E-03	1.42E-05
TM5ΔV1-3	1.06E+07	6.86E+03	4.74E+01	6.50E-04	4.84E-06
**gl NIH45-46**					
TM1	6.29E+05	9.63E+03	4.56E+02	1.53E-02	2.10E-04
TM2	1.45E+05	1.84E+04	5.15E+03	1.27E-01	1.02E-02
TM3	2.17E+05	2.90E+04	8.23E+03	1.34E-01	9.20E-03
TM4	3.41E+05	1.86E+04	2.00E+03	5.46E-02	1.86E-03
TM5	4.74E+06	1.53E+04	1.83E+02	3.23E-03	1.27E-05
TM1ΔV1-3	6.82E+05	4.05E+03	4.15E+01	5.94E-03	1.41E-05
TM4ΔV1-3	2.11E+05	4.78E+03	2.34E+02	2.26E-02	2.84E-04
TM5ΔV1-3	4.59E+06	6.23E+03	3.81E+01	1.36E-03	4.70E-06
**gl 12A21**					
TM1	-	-	-	-	-
TM2	2.01E+05	5.52E+04	1.15E+04	2.74E-01	2.12E-02
TM3	1.74E+05	4.85E+04	9.76E+03	2.79E-01	1.67E-02
TM4	3.34E+05	5.62E+04	5.35E+03	1.68E-01	5.24E-03
TM5	3.85E+04	1.55E+04	6.15E+03	4.03E-01	2.34E-02
TM1ΔV1-3	-	-	-	-	-
TM4ΔV1-3	3.11E+05	9.47E+03	8.13E+02	3.05E-02	6.14E-04
TM5ΔV1-3	3.08E+04	1.59E+03	9.83E+02	5.15E-02	1.72E-03
**gl VRC-PG19**					
TM1	2.87E+05	4.00E+04	4.20E+03	1.40E-01	4.93E-03
TM2	4.16E+05	7.15E+04	1.17E+04	1.72E-01	7.03E-03
TM3	2.24E+06	9.89E+04	3.99E+03	4.42E-02	5.63E-04
TM4	5.45E+06	9.80E+04	4.21E+03	1.80E-02	1.65E-04
TM5	5.22E+06	8.38E+04	3.36E+03	1.60E-02	1.48E-04
TM1ΔV1-3	3.53E+05	1.11E+04	3.29E+02	3.15E-02	2.48E-04
TM4ΔV1-3	8.90E+06	2.41E+04	1.54E+02	2.70E-03	4.92E-06
TM5ΔV1-3	8.93E+06	1.93E+04	1.09E+02	2.16E-03	4.04E-06

Env Name	K_A(M-1)	k_{on} ($M^{-1}s^{-1}$)	k_{on} error	k_{off} (s^{-1})	k_{off} error
**glVRC-PG20**					
TM1	1.40E+07	4.97E+04	9.74E+02	3.43E-03	1.39E-05
TM2	3.88E+06	5.09E+04	1.54E+03	1.29E-02	9.98E-05
TM3	1.20E+07	6.05E+04	1.28E+03	4.87E-03	1.92E-05
TM4	2.28E+07	6.11E+04	1.27E+03	2.57E-03	1.11E-05
TM5	1.78E+08	1.12E+05	1.13E+03	5.53E-04	5.05E-06
TM1ΔV1-3	1.24E+07	1.35E+04	1.99E+02	1.04E-03	9.40E-06
TM4ΔV1-3	3.84E+07	2.45E+04	1.11E+02	5.11E-04	3.11E-06
TM5ΔV1-3	2.45E+08	4.12E+04	1.46E+02	1.49E-04	2.35E-06
**gl3BNC60**					
TM1	-	-	-	-	-
TM2	-	-	-	-	-
TM3	-	-	-	-	-
TM4	-	-	-	-	-
TM5	-	-	-	-	-
TM1ΔV1-3	-	-	-	-	-
TM4ΔV1-3	3.49E+03	1.41E+03	2.31E+03	3.98E-01	3.12E-02
TM5ΔV1-3	2.18E+03	1.04E+03	3.31E+03	4.77E-01	4.79E-02
**gl VRC-CH31**					
TM1	-	-	-	-	-
TM2	-	-	-	-	-
TM3	-	-	-	-	-
TM4	-	-	-	-	-
TM5	-	-	-	-	-
TM1ΔV1-3	-	-	-	-	-
TM4ΔV1-3	-	-	-	-	-
TM5ΔV1-3	8.95E+03	2.40E+03	4.17E+03	2.40E-01	1.65E-02

Values are the average of at least 2 serial Fab dilutions giving a R^2 value of > 0.95. (-): no detectable binding.

Supplementary Table 2. Summary of gl3BNC60 sequences isolated from antigen-specific IgG^{+} B cells post immunization

Heavy Chain Sequences		
# of sequences	nt substitutions	AA substitutions
10	0	0
1	3	1
1	1	1
**Light Chain Sequences**		
57	0	0
1	2	0
1	1	1
1	3	3
1	11	8

Supplementary Table 3. Neutralization of HIV-1 pseudoviruses by sera from immunized mice.

	ID50 Titer in TZM.bl cells (1/x)							
Sample ID	MuLV	426C WT	426C.TM1	426C.TM4	426C.S278A. T462A. T465A	T247-23*	62357.14*	426c.TM4 ΔV1-3 immunogen
ES15	<10	<10	<10	<10	<10	<10	<10	Heptamer
HR18	<10	<10	<10	<10	<10	<10	<10	Heptamer
HR19	<10	<10	<10	**24**	<10	<10	<10	Heptamer
ES3D	<10	<10	<10	<10	<10	<10	<10	gp120-Dex
ES4D	<10	<10	<10	<10	<10	<10	<10	gp120-Dex
ES5D	<10	<10	<10	<10	<10	<10	<10	gp120-Dex
HS18	<10	<10	<10	<10	<10	<10	<10	Ferritin
HS19	<10	<10	<10	<10	<10	<10	<10	Ferritin
ES26	<10	<10	<10	<10	<10	<10	<10	Ferritin
ES27	<10	<10	<10	<10	<10	<10	<10	Ferritin
WT Pool	<10	<10	<10	**18**	<10	<10	<10	Ferritin
Naïve gl3BNC60 KI	<10	<10	<10	<10	<10	<10	<10	None

*Viruses naturally lack an NLGS at position 276
